# Intuitionistic Fuzzy Synthetic Measure on the Basis of Survey Responses and Aggregated Ordinal Data

**DOI:** 10.3390/e23121636

**Published:** 2021-12-06

**Authors:** Bartłomiej Jefmański, Ewa Roszkowska, Marta Kusterka-Jefmańska

**Affiliations:** 1 Department of Econometrics and Computer Science, Wroclaw University of Economics and Business, 53-345 Wrocław, Poland; 2 Faculty of Computer Science, Bialystok University of Technology, Wiejska 45A, 15-351 Bialystok, Poland; e.roszkowska@pb.edu.pl; 3 Department of Quality and Environmental Management, Wroclaw University of Economics and Business, 53-345 Wrocław, Poland; marta.kusterka-jefmanska@ue.wroc.pl

**Keywords:** synthetic measure, fuzzy measurement, ordinal data, intuitionistic fuzzy set, uncertainly, decision making, fuzzy multi-criteria method, Hellwig’s method

## Abstract

The paper addresses the problem of complex socio-economic phenomena assessment using questionnaire surveys. The data are represented on an ordinal scale; the object assessments may contain positive, negative, no answers, a “difficult to say” or “no opinion” answers. The general framework for Intuitionistic Fuzzy Synthetic Measure (IFSM) based on distances to the pattern object (ideal solution) is used to analyze the survey data. First, Euclidean and Hamming distances are applied in the procedure. Second, two pattern object constructions are proposed in the procedure: one based on maximum values from the survey data, and the second on maximum intuitionistic values. Third, the method for criteria comparison with the Intuitionistic Fuzzy Synthetic Measure is presented. Finally, a case study solving the problem of rank-ordering of the cities in terms of satisfaction from local public administration obtained using different variants of the proposed method is discussed. Additionally, the comparative analysis results using the Intuitionistic Fuzzy Synthetic Measure and the Intuitionistic Fuzzy TOPSIS (IFT) framework are presented.

## 1. Introduction

Multiple criteria decision making (MCDM) has been an important research discipline of decision science applied in many areas such as business, management, engineering, and social science [1,2,3]. Nowadays, a lot of new MCDM methods have been introduced to address several practical problems and real-life applications. MCDA methods are widely used in constructing synthetic measures (or composite indicators) for the evaluation of complex socio-economic phenomena [4,5,6].

One of the problems is the assessment of complex socio-economic phenomena using questionnaire surveys when data are represented on an ordinal scale, especially if the object assessments contain positive, negative opinions and an element of uncertainty expressed in the form of no answer, “difficult to say” answer, “no opinion”, etc. In previous studies, some proposals of TOPSIS and Hellwig’s methods based on intuitionistic fuzzy numbers to solve the presented problems were discussed.

The classical Hellwig’s method was presented in 1968 by a Polish researcher as a taxonomic method for international comparison of economic development of countries [7]. This method allows ranking multidimensional objects in terms of a complex phenomenon that cannot be described using a single criterion. The method is based on the concept of distance from the pattern object, which was also used in the well-known and popular TOPSIS method. The difference between both methods is that TOPSIS, apart from the distance from the pattern object, also takes into account the distance from the anti-pattern object.

The methods based on Hellwig’s and TOPSIS methodology have many features in common, whereas the main difference concerns the method for calculating the synthetic variable value. The methods are characterized by the simplicity of calculations and software options (e.g., available free R packages). Both methods allow including quantitative and qualitative criteria in the assessment of objects. In the case of quantitative criteria, their normalization is required. Fuzzy modifications of both methods were proposed for the qualitative criteria. They consist in replacing the qualitative criteria values with fuzzy sets (most frequently fuzzy numbers). In the vast majority of cases the parameters of fuzzy numbers are determined subjectively by the researchers, which does not always allow reflecting the respondents’ preferences in this matter. It is also worth noting that both methods do not suggest how to determine the weighting factors for the criteria. In addition, they do not take into account the potential correlations between the criteria.

Hellwig’s method was promoted in the world literature through the UNESCO research project on the human resources indicators for less developed countries [8,9]. Another application of this method can be found, e.g., in the studies [10,11,12,13,14]. Hellwig’s method was also extended for the fuzzy environment [15,16,17], the intuitionistic fuzzy environment [18,19,20] and the interval-valued intuitionistic fuzzy environment [21].

The idea of using MCDM methods in measuring complex phenomena based on survey data is quite new, therefore the source literature offers only a few scientific publications and research studies addressing this area. The paper [18] presents the concept of the IFSM using Hellwig’s approach for the intuitionistic fuzzy sets. The IFSM allows measuring complex phenomena based on the respondents’ opinions. The IFSM adopts that the respondents assess objects in terms of the adopted criteria using ordinal measurement scales. The respondents’ opinion measurement results are later transformed into intuitionistic fuzzy sets. In another paper [21] a synthetic measure based on Hellwig’s approach and the interval-valued intuitionistic fuzzy set theory is presented. Also, the optimism coefficient is defined, which allows setting the limits of intervals for the proposed parameters. The common feature of both methods is using the transformation of ordinal data to the form of intuitionistic fuzzy sets. The assessment criteria are thus expressed in the form of three parameters of the intuitionistic fuzzy set: membership, non-membership and uncertainty. The difference in these methods consists in determining values of these parameters. In the first case (IFSM method) they are presented as numbers in the interval [0, 1], while in the second case (I-VIFSM method) they take the form of intervals. Finally, [19] proposed the Intuitionistic Fuzzy TOPSIS (IF-TOPSIS) method which can be applied for assessing socio-economic phenomena on the basis of survey data.

Motivated by the above-mentioned works, the present paper proposes the general framework for intuitionistic fuzzy multi-criteria procedure, namely the Intuitionistic Fuzzy Synthetic Measure (IFSM) based on distance to the pattern object. The IFSM method has been inspired by Hellwig’s approach of developing a coefficient adapted to an intuitionistic fuzzy environment.

The Intuitionistic Fuzzy Synthetic Measure was proposed to address the problem of survey data. It consists of seven main steps: (1) representation of the survey data in the form of intuitionistic fuzzy values; (2) determination of the Intuitionistic Fuzzy Decision Matrix; (3) determination of the intuitionistic fuzzy pattern object; (4) calculation of the distance measures; (5) calculation of the intuitionistic fuzzy coefficient; (6) rank ordering of objects by maximizing the coefficient; and (7) comparing the criteria with the Intuitionistic Fuzzy Synthetic Measure.

Two concepts for determining intuitionistic fuzzy pattern objects are discussed. The first one is based on max values from the survey data and the second on max intuitionistic values in general. Next, two measures of distances, i.e., Euclidean and Hamming distance implemented in the coefficient procedure are considered which, additionally, can be based on two or three parameters. This provides eight variants of the proposed IFSM. The usefulness of the proposed approach was examined in the evaluation of satisfaction from local public administration in the context of quality of life in cities using survey data.

As was pointed out by [22] the purpose of constructing synthetic measure, among other things, “to condense and summarise the information contained in a number of underlying indicators, in a way that accurately reflects the underlying concept”. Thus finally, the Spearman coefficient for comparison criteria with respect to information transferred for the IFSM is proposed.

The objectives and contributions of this study are presented below:

- to develop a general IFSM based on Hellwig’s approach for the evaluation of socio-economic phenomena with survey data;
- to study the IFSM based on Hellwig’s approach taking into account two types of Euclidean and Hamming distance implemented in the procedure;
- to study the IFSM based on Hellwig’s approach considering two or three parameters used in distance measure applied in the procedure;
- to study the IFSM based on Hellwig’s approach examining different pattern object construction used in the procedure;
- to propose the method for criteria comparison with the Intuitionistic Fuzzy Synthetic Measure;
- to demonstrate different variants of the Intuitionistic Synthetic Measure based on Hellwig’s approach through a comparative analysis;
- to compare different variants of the IFSM based on Hellwig’s approach with the Intuitionistic Fuzzy TOPSIS (IFT) procedure to examine its relevance and effectiveness.

The proposed framework, based on the extended Hellwig’s method, has been applied to analyse its relevance.

The rest of this article is organized as follows. In Section 2 the basic concepts related to intuitionistic fuzzy sets (IFS) and distances on IFSs are presented. The general framework of the IFSM based on Hellwig’s approach is provided in Section 3. Section 4 discusses a case study solving the problem of rank-ordering of the cities in terms of satisfaction from local public administration using the proposed approach. The comparison results obtained using the IFSM with the IFT framework are also presented. The conclusion and indications for future research are formulated in Section 4.

## 2. Preliminaries

To start with, the presentation of some basic concepts related to IFS and distances on IFSs are presented.

The Intuitionistic Fuzzy Set theory, proposed by Atanassov [23], is an extension of the Fuzzy Set (FS) theory introduced by Zadeh [24] to address uncertainty.

**Definition** **1** ([23,25])**.** *Let* X *be a universe of discourse of objects. An intuitionistic fuzzy set* A in X *is given by:*

(1) $$A=\left\{<x,\;\mu_{A}(x),\;\nu_{A}(x)>\left|x\in X\right.\right\}\;$$

*where* $\mu_{A},\;\nu_{A}:X\to [0,\;1]$ *are functions with the condition for every* x∈X

(2) $$0\leq \mu_{A}(x)+\nu_{A}(x)\leq 1$$

The numbers $\mu_{A}(x)$ and $\nu_{A}(x)$ denote, respectively, the degrees of membership and non-membership of the element x∈X to the set A; $\pi_{A}(x)=1-\mu_{A}(x)-\nu_{A}(x)$ the intuitionistic fuzzy index (hesitation margin) of the element x in the set A. Greater $\pi_{A}(x)$ indicates more vagueness. It should be noticed that when $\pi_{A}(x)=0$ for every x∈X then intuitionistic fuzzy set A is an ordinary fuzzy set.

If the universe X contains only one element x, then the IFS over X is denoted as $A=(\mu_{A},\;\nu_{A})$ and called an intuitionistic fuzzy value (IFV) [26,27]. Let Θ be the set of all IFVs. The intuitionistic value $\left(1,0\right)$ is the largest, while $\left(0,1\right)$ is the smallest.

One of the applications of intuitionistic fuzzy sets in multiple criteria decision making is the possibility of taking into consideration the decision maker’s approval, rejection, and hesitations regarding the evaluated alternatives with respect to criteria. This is the main motivation for using the intuitionistic fuzzy sets in developing the multi-criteria procedure.

Euclidean and Hamming distances represent the widely used distances for the intuitionistic fuzzy sets [28].

**Definition** **2** ([28])**.** *Let us consider two* A, B∈IFS *with membership functions* $\mu_{A}(x)$, $\mu_{B}(x)$, *and non-membership functions* $\nu_{A}(x)$, $\nu_{B}(x)$, *respectively. The normalized Euclidean between two intuitionistic fuzzy sets A and B is defined as:*

(3) $$e_{IFS}^{2}(A,\;B)=\sqrt{\frac{1}{2n}\sum_{j=1}^{n}\left[{\left(\mu_{A}(x_{i})-\mu_{B}(x_{i})\right)}^{2}+{\left(\nu_{A}(x_{i})-\nu_{B}(x_{i})\right)}^{2}\right]}\;$$

(4) $$e_{IFS}^{3}(A,\;B)=\sqrt{\frac{1}{2n}\sum_{j=1}^{n}\left[{\left(\mu_{A}(x_{i})-\mu_{B}(x_{i})\right)}^{2}+{\left(\nu_{A}(x_{i})-\nu_{B}(x_{i})\right)}^{2}+{\left(\pi_{A}(x_{i})-\pi_{B}(x_{i})\right)}^{2}\right]}$$

The normalized Hamming distance between two intuitionistic fuzzy sets *A* and *B* is defined as:

(5) $$h_{IFS}^{2}(A,\;B)=\frac{1}{2n}\sum_{j=1}^{n}\left[\left|\mu_{A}(x_{i})-\mu_{B}(x_{i})\right|+\left|\nu_{A}(x_{i})-\nu_{B}(x_{i})\right|\right]$$

(6) $$h_{IFS}^{3}(A,\;B)=\frac{1}{2n}\sum_{j=1}^{n}\left[\left|\mu_{A}(x_{i})-\mu_{B}(x_{i})\right|+\left|\nu_{A}(x_{i})-\nu_{B}(x_{i})\right|+\left|\pi_{A}(x_{i})-\pi_{B}(x_{i})\right|\right]$$

To compare two IFVs the following score function defined by Chen & Tan [29] was used:

(7) $$Sc(A)=\mu_{A}-\nu_{A}$$

and accuracy function defined by Hong and Choi [30]:

(8) $$H(A)=\mu_{A}+\nu_{A}$$

It can be easily observed that Sc(A)∈[−1, 1] and H(A)∈[0, 1].

**Definition** **3** ([31])**.** *Let us consider two intuitionistic fuzzy values* $A=(\mu_{A},\;\nu_{A})$, $B=(\mu_{B},\;\nu_{B})$, *respectively:*

*1*. *if Sc(A)<Sc(B), then A<B;*
*2*. *if Sc(A)=Sc(B), and*
  *(i)*  *H(A)<H(B), then A<B;*
  *(ii)*  *H(A)=H(B), then A=B.*

## 3. Classical and Intuitionistic Variant of Hellwig’s Method

### 3.1. Classical Variant of Hellwig’s Method

The classical Hellwig’s method was proposed for quantitative criteria. It adopts the calculation of Euclidean distance from the pattern of development for each assessed object. Most often the pattern of development is an abstract unit presenting the most favorable assessments of the individual criteria. Let $O=\left\{O_{1},\;O_{2},\;\ldots ,\;O_{m}\right\}$ i=1, 2, …, m be the set of objects subject to assessment and $C=\left\{C_{1},\;C_{2},\;\ldots ,\;C_{n}\right\}$ j=1, 2, …, n the set of criteria constituting a complex phenomenon. It should also be adopted that P and N are the sets of stimulating (positive) and destimulating (negative) criteria, respectively, influencing the complex phenomenon (C=P∪N). The classical variant of Hellwig’s method consists of the following steps:

**Step 1.** Defining the decision matrix:

(9) $$D=[x_{ij}]$$

where $x_{ij}$ is the value of the i-th object with respect to the j-th criterion.

**Step 2.** Determining the normalized decision matrix:

(10) $$Z=[z_{ij}]$$

using the formula for standardization:

(11) $$z_{ij}=\frac{x_{ij}-{\bar{x}}_{j}}{S_{j}}$$

where: ${\bar{x}}_{ij}=\frac{1}{m}\sum_{i=1}^{m}x_{ij}$, $S_{j}=\sqrt{\frac{1}{m}\sum_{i=1}^{m}{\left(x_{ij}-{\bar{x}}_{ij}\right)}^{2}}$.

**Step 3**. Defining the pattern of development (pattern object) $O^{+}=\left[z_{1}^{+},\;z_{2}^{+},\;\ldots ,\;z_{n}^{+}\right]$ in accordance with the principle:

(12) $$z_{j}^{+}=\left\{\begin{array}{c} \max z_{ij}\;if\;z_{ij}\in P\\ \min z_{ij}\;if\;z_{ij}\in N \end{array}\right.$$

**Step 4**. Calculating the distance of the i-th object from the pattern of development using the Euclidean distance:

(13) $$d_{i}^{+}=\sqrt{{\sum_{j=1}^{n}\left(z_{ij}-z_{j}^{+}\right)}^{2}}$$

**Step 5**. Calculating the synthetic measure of development for the i-th object:

(14) $$H_{i}=1-\frac{d_{i}^{+}}{d_{0}}$$

where: $d_{0}=\bar{d}+2S$, $\bar{d}=\frac{1}{n}\sum_{i=1}^{m}d_{i}^{+}$, $S=\sqrt{\frac{1}{n}\sum_{i=1}^{m}{\left(d_{i}^{+}-\bar{d}\right)}^{2}}$.

**Step 6**. Ranking the objects according to the decreasing values of $H_{i}$.

The measure most often takes values from the interval [0, 1]. The higher values of the measure the less the object is away from the pattern of development.

### 3.2. The Intuitionistic Fuzzy Synthetic Measure Based on Hellwig’s Approach for the Evaluation of Socio-Economic Phenomena Using Survey Data

In this section, a general framework for Intuitionistic Fuzzy Synthetic Measures is proposed. Let $O=\left\{O_{1},\;O_{2},\;\ldots ,\;O_{m}\right\}$ i=1, 2, …, m be the set of objects under the survey evaluation, $C=\left\{C_{1},\;C_{2},\;\ldots ,\;C_{n}\right\}$ j=1, 2, …, n the set of criteria for the objects assessed by the respondents using an ordinal measurement scale. The respondents’ answers are collected in a questionnaire survey. It was adopted that the respondents answered the questions using different scales, which can be aggregated into three groups: “a positive opinion about the object”, “a negative opinion about the object”, “no opinion or no answer”. The same importance was adopted in the evaluation of objects to the criteria. i.e., the weights of criteria are equal [32].

The procedure to evaluate the socio-economic phenomena is as follows:

**Step 1**. Representation of the survey data in the form of intuitionistic fuzzy values.

The respondents’ opinions about the object $O_{i}$ for each criterion $C_{j}$ are represented by IFVs $\left(\mu_{ij},\;\nu_{ij}\right)$, where:

- $\mu_{ij}$—the fraction of positive opinions about *i*-th object with respect to *j*-th criterion,
- $\nu_{ij}$—the fraction of negative opinions about *i*-th object with respect to *j*-th criterion,
- $\pi_{ij}$—the fraction of opinion type “don’t know”, “no answers” for *i*-th object with respect to *j*-th criterion, and $\pi_{ij}(x)=1-\mu_{ij}(x)-\nu_{ij}(x)$.

The following has been adopted:

(15) $$\mu_{ij}=\frac{p_{ij}}{N_{ij}},\nu_{ij}=\frac{n_{ij}}{N_{ij}},\pi_{ij}=\frac{h_{ij}}{N_{ij}},$$

where:

- $p_{ij}$—the total number of respondents who positively evaluated the *i*-th object with respect to *j*-th criterion;
- $n_{ij}$—the total number of respondents who negatively evaluated the *i*-th object with respect to *j*-th criterion;
- $h_{ij}$—the total number of respondents with hesitancy opinion about the *i*-th object for *j*-th criterion;
- $N_{ij}$—the total number of respondents who evaluated the *i*-th object with respect to the *j*-th criterion.

It has been noted that $p_{ij}+n_{ij}+h_{ij}=N_{ij}$.

Clearly, instead of the total number of responses, the percentage of relevant responses common for the secondary survey data can be used.

In this way *i*-th object $O_{i}$ is represented by the vector:

(16) $$O_{i}=[(\mu_{i1},\;\nu_{i1}),\;\ldots ,\;(\mu_{in},\;\nu_{in})]$$

where i=1, 2, …, m.

**Step 2.** Determination of the Intuitionistic Fuzzy Decision Matrix.

Based on the survey data representation in the form of intuitionistic fuzzy values obtained in step 1 the intuitionistic fuzzy decision matrix is given in the form:

(17) $$D=\left[\begin{array}{cccc} \left(\mu_{11},\;\nu_{11}\right) & \left(\mu_{12},\;\nu_{12}\right) & \ldots & \left(\mu_{1n},\;\nu_{1n}\right)\\ \left(\mu_{21},\;\nu_{21}\right) & \left(\mu_{22},\;\nu_{22}\right) & \ldots & \left(\mu_{2n},\;\nu_{2n}\right)\\ \ldots & \ldots & \ldots & \ldots \\ \left(\mu_{m1},\;\nu_{m1}\right) & \left(\mu_{m1},\;\nu_{m1}\right) & \ldots & \left(\mu_{mn},\;\nu_{mn}\right) \end{array}\right]$$

**Step 3.** Determination of the intuitionistic fuzzy pattern object.

The intuitionistic fuzzy pattern object $\left(I_{IFI}\right)$ can be determined twofold:

- is based on maximum *IFV* and takes the form of:
  (18) $$I_{IFI}^{1}=[(1,\;0),\;\ldots ,\;(1,\;0)]\;$$
- is based on maximum and minimum values and takes the form of:
  (19) $$I_{IFI}^{2}=[(\underset{i}{\max}\mu_{i1},\;\underset{i}{\min}\nu_{i1}),\;\ldots ,\;(\underset{i}{\max}\mu_{in},\;\underset{i}{\min}\nu_{in})]\;$$
  where $\left(\mu_{ij},\;\nu_{ij}\right)$, denote the evaluation information of *i*-th object with respect to *j*-th criterion and $\pi_{ij}=1-\mu_{ij}(x)-\nu_{ij}(x)$.

**Step 4.** Calculation of the distance measures.

After selecting the distance measure, the distance measures between the objects and the intuitionistic fuzzy pattern object selected in step 3 are calculated using one of the Formulas (3)–(6).

The distance measure from the pattern object takes the form of:

(20) $$d^{+}(O_{i})=d(I_{IFS},\;O_{i})$$

where $I_{IFS}\in \left\{I_{IFS}^{1},\;I_{IFS}^{2}\right\}$, $d\in \left\{e_{IFS}^{3},\;e_{IFS}^{2},\;h_{IFS}^{2},\;h_{IFS}^{3}\right\}$.

**Step 5.** Calculation of the Intuitionistic Fuzzy Synthetic Measure.

The Intuitionistic Fuzzy Synthetic Measure (IFSM) coefficient is defined as follows:

(21) $$IFSM(O_{i})=1-\frac{d^{+}(O_{i})}{d_{0}}$$

where: $d_{0}={\bar{d}}_{0}+2S(d_{0})$, ${\bar{d}}_{0}=\frac{1}{n}\sum_{i=1}^{n}d^{+}(O_{i})$, $S(d_{0})=\sqrt{\frac{1}{n}\sum_{i=1}^{n}\left(d^{+}(O_{i}\right)-{\bar{d}}_{0}{)}^{2}}$.

**Step 6.** Rank ordering of objects by maximizing the coefficient $IFSM(O_{i})$.

The highest value of $IFSM(O_{i})$ then the highest position of the object $O_{i}$.

**Step 7.** Comparing the individual criteria with the Intuitionistic Fuzzy Synthetic Measure using Information Transfer Measure (ITM).

The important two problems should be addressed while building the IFSM, condensing information and accurately representing the underlying concept. The criteria should capture the most important properties of the analyzed phenomena, represent them accurately and provide a large amount of information. There should be a positive correlation between the criteria and the synthetic measure, and also each criterion should contribute to the decision-maker(s)’ views on its importance regarding the concept [22]. Now the measure of the information transferred from each criterion to the IFSM is defined. The criteria should capture the most important properties of the analyzed phenomena, represent them accurately and provide a large amount of information.

First, the individual criteria represented by the intuitionistic fuzzy values are ordered using accuracy function and score function (see Definition 3). Then the Spearman coefficient between the ranking criteria and the ranking obtained by the IFSM measure is calculated. The Spearman coefficient is a nonparametric measure of dependence for the variables measured at least on an ordinal scale. The measure is normalized in the range [–1, 1]. It allows measuring the power and determining the direction of the correlations. Formally, the Information Transfer Measure for *j*-th criterion is defined as follows:

(22) $$ITM_{j}=\mathrm{Spearman}\;\mathrm{coefficient}(\mathrm{rank}\;C_{j},\;\mathrm{rank}\;IFSM)$$

The $ITM_{j}$ shows the power and direction between the criterion $C_{j}$ and the synthetic measure IFSM. It should be observed that taking into account the way of survey data representation in the form of intuitionistic fuzzy values this coefficient should be positive. In the case where the importance of the criterion is the same, the measures $ITM_{j}$ for j=1, 2, …, n should be similar.

**Figure 1:**
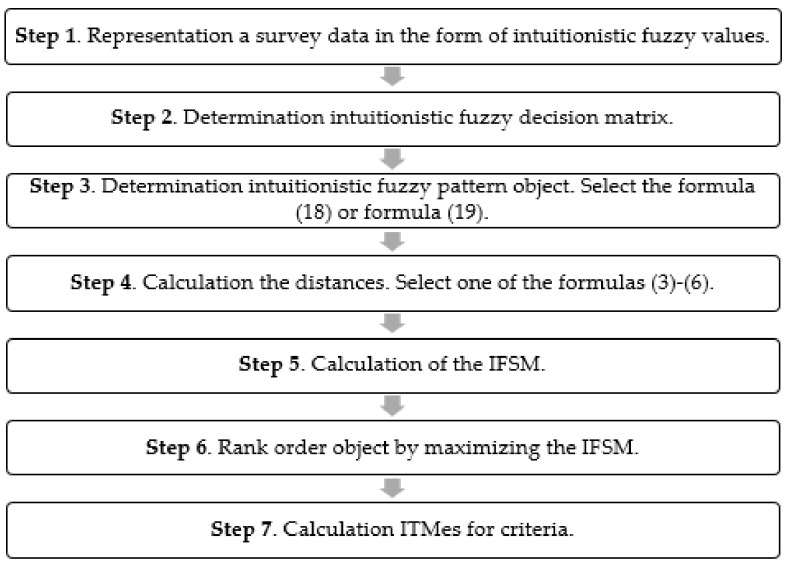
Procedure for the analysis of survey data for IFSM.

The procedure of analyzing the survey data for IFSM is presented in Figure 1.

Classification of variants of the IFSM based on an intuitionistic fuzzy framework with respect to pattern objects and distance measures is presented in Table 1.

## 4. Empirical Example

### 4.1. Problem Description and Data Source

The approach to the analysis of survey data proposed in the article, applying the presented procedure and the IFSM method, was used in the analysis of the results from the fifth survey on quality of life in European cities. The survey provides a unique insight into city life. It gathers the experience and opinions of city dwellers.

The fifth survey on quality of life in European cities was conducted for the European Commission by the IPSOS company. The survey covered the inhabitants of 83 cities in the EU, the EFTA countries, the UK, the Western Balkans, and Turkey. The survey was conducted between 12 June and 27 September 2019, with a break between 15 July and 1 September. A total of 700 interviews were completed in each surveyed city. This means that a total of 58,100 inhabitants of 83 cities participated in the survey.

The survey covers eight fields of the quality of life in cities: overall satisfaction, services and amenities, environmental quality, economic well-being, public transport, the inclusive city, local public administration, as well as safety and crime. For the first time, the fifth round of the survey includes questions about the quality of the city administration. The high-quality, efficient, and transparent local public administration is very important for improving the quality of life in European cities. In addition, improving the quality of institutions at the local level is the heart of the EU and the EU Cohesion Policy. In the empirical example, European cities would be ranked only in the field of local public administration. Thus, only five questions of the questionnaire concerning satisfaction from the local public administration were used [33]:

“I will read you a few statements about the local public administration in your city. Please tell me whether you strongly disagree, somewhat disagree,…

Q1: I am satisfied with the amount of time it takes to get a request solved by my local public administration;

Q2: The procedures used by my local public administration are straightforward to understand;

Q3: The fees charged by my local public administration are reasonable;

Q4: Information and services of my local public administration can be easily accessed online;

Q5: There is corruption in my local public administration.”

Our study aims at measuring and benchmarking inhabitants’ satisfaction with local public administration using the IFSM approach. Inhabitants’ satisfaction, as a complex phenomenon, was characterized using five criteria described by five questions Q1–Q5: C_1_—time for request, C_2_—procedures; C_3_—fees charged, C_4_—information and services, C_5_—corruption. In the assessment of criteria, a five-point measurement scale was used: strongly disagree, somewhat disagree, somewhat agree, strongly agree, don’t know/no answer.

The characteristics of the research sample in terms of gender, age, and level of education are presented in Table 2.

### 4.2. Analysis of the Results

In this part, the empirical results concerning the evaluation of the satisfaction with local public administration in European cities using the IFSMes are presented. Due to the large number of cities covered by the survey individual steps of the proposed procedure were presented based on the example of the selected 2 cities: Zurich (the best in all rankings) and Palermo (the worst in all rankings). The selected cities were the first and the last in the ranking obtained using all IFSM methods. The assessment of the selected cities in terms of 5 criteria using the 5 categories is presented in Table 3.

According to Formula (15), the respondents’ assessments were transformed into IFVs (Table 4).

It has been observed that for the criteria C_1_, C_2_, C_3_, C_4_ the ν is obtained by summing up the categories 1, 2, and μ by summing up the categories 3, 4. Taking into account the form of question Q5 for the criterion C_5_ the ν value is obtained by summing up the categories 3, 4 while μ by summing up the categories 1, 2. The assessment criteria in the form of IFVs for all cities are listed in Table A1, Table A2 and Table A3 in the Appendix A.

The assessments of cities in terms of five criteria in the form of IFVs were used to construct an intuitionistic fuzzy decision matrix, a fragment of which is presented below for the three selected cities:

$$\begin{array}{cc} & C_{1}\;\;\;\;\;C_{2}\;\;\;\;\;C_{3}\;\;\;\;\;C_{4}\;\;\;\;\;C_{5}\\ \begin{array}{r} \mathrm{Aalborg}\\ \ldots \\ D=\mathrm{Palermo}\\ \ldots \\ \mathrm{Zurich} \end{array} & \left[\begin{array}{ccccc} \left(0.164,\;0.670\right) & \left(0.293,\;0.608\;\right) & \left(0.246,\;0.568\right) & \left(0.101,\;0.846\right) & \left(0.166,\;0.788\right)\\ \ldots & \ldots & \ldots & \ldots & \ldots \\ \left(0.836,\;0.128\right) & \left(0.689,\;0.280\right) & \left(0.765,\;0.221\right) & \left(0.450,\;0.510\right) & \left(0.719,\;0.206\right)\\ \ldots & \ldots & \ldots & \ldots & \ldots \\ \left(0.124,\;0.733\right) & \left(0.206,\;0.723\right) & \left(0.188,\;0.771\right) & \left(0.083,\;0.801\right) & \left(0.171,\;0.672\right) \end{array}\right] \end{array}$$

Weights have not been assigned to individual criteria. In our opinion, all the aspects (e.g., time for request, procedures, fees charged, information and services, corruption) should be balanced, i.e., they are equally important in evaluating satisfaction from the local administration.

The coordinates of intuitionistic fuzzy pattern objects were determined twofold: based on (1,0) values and second for maximum and minimum IFVs, respectively (Table 5 and Table 6).

Using the normalized Euclidean or Hamming distance in accordance with the Formulas (3)–(6) the distances $d^{+}$ of each city from the intuitionistic fuzzy pattern objects and $d_{0}$ values were calculated. Finally, the IFSM coefficients were calculated (Table 7).

The values of IFSM coefficients for all cities are presented in Table A4 in the Appendix A.

The position of cities in the ranking was determined based on the IFSM coefficient values, following the principle that the higher the value of the IFSM coefficient, the higher the city’s position in the ranking (Table A5; Appendix A).

**Figure 2:**
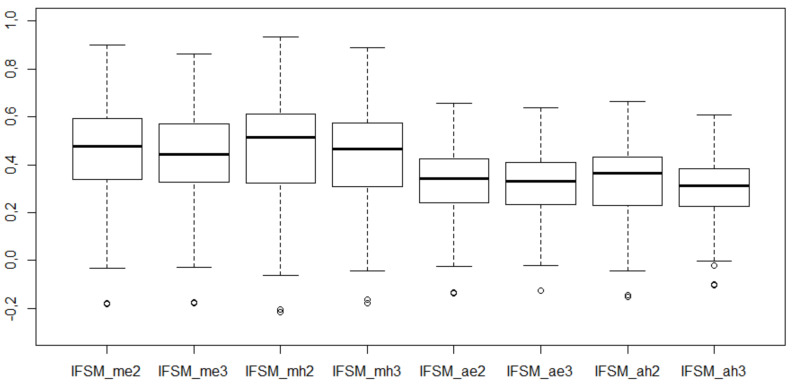
Box plots for the IFSM values.

Descriptive statistics and box plots for the values of IFSM coefficients are presented in Table 8 and Figure 2.

Based on Table 8 and Figure 2 three main observations can be made:

- determining the coordinates of the pattern object based on intuitionistic values (1,0) resulted in a lower value of the IFSM for cities compared with the IFSM when the coordinates of the pattern object are based on max and min values. The IFSM area of variability also decreased;
- the IFSM similarly differentiates cities in terms of the adopted synthetic criterion, i.e., satisfaction with public administration services; regardless of the method used for determining the coordinates of the pattern object, the number of cities for which the IFS values are below and above the IFSM average remains at a similar level;
- regardless of the method for determining the coordinates of the pattern object, the IFSM values present a slight response to the choice of the distance measure and the number of parameters that take these distances into account. The introduction of the third uncertainty parameter in measuring the distance between cities and the pattern object slightly lowers the mean values of IFSM and reduces the variability range of these values. This regularity has been observed for two methods used in determining the coordinates of the pattern object.

The Spearman coefficients between IFSM values are presented in Table 9.

The choice between the Euclidean and Hamming distance and the method for determining the coordinates of the pattern objects does not have a large impact on the ranking positions of the cities. High values of the Spearman coefficient suggest slight changes in the ranking position of the cities. If the Hamming distance for two parameters is used in IFSM, the choice of the method for determining the coordinates of the pattern objects is irrelevant. In both cases, the value of the Spearman coefficient was equal to one, which means the same ranking of the cities in terms of satisfaction with public administration services. The lowest similarity of rankings (Spearman coefficient value equal to 0.870) was observed for the IFSM with the Hamming distance with two and three parameters, respectively, for the coordinates of the pattern objects determined based on the values (max, min) and (1,0).

The Information Transfer Measures for the IFSMes are presented in Table 10.

The criteria are well represented by the IFSM. The largest information transfer occurred for C_1_ criterion (regardless of the IFSM variant). The smallest information transfer was recorded for C_3_ criterion. All the criteria are the least represented by the IFSM__ae3_ variant. The same values of Spearman coefficients were observed for the two variants: IFSM__mh2_ and IFSM__ah2_. This result is not surprising since equal rankings were obtained using these variants of the methods (see Table 10).

### 4.3. Comparative Analysis and Implications

Hellwig’s method uses only the concept of a positive pattern object (named as the pattern of development), while the well known TOPSIS method [34] used the concept of pattern and anti-pattern object (ideal and anty-ideal solution, respectively). The TOPSIS with many modifications in the fuzzy and intuitionistic fuzzy environment has been proposed and applied in real-life problems [19,35,36].

Similarly, a description of variants of the IFTes with respect to pattern objects and distance measures used in comparative analysis is presented in Table 11 (for details see [19]).

The coordinates of an intuitionistic fuzzy anti-pattern object based on (0,1) values or max and min in the IFT are presented in Table 12. The coordinates of an intuitionistic fuzzy anti-pattern object based on max and min values are presented in Table 13.

**Figure 3:**
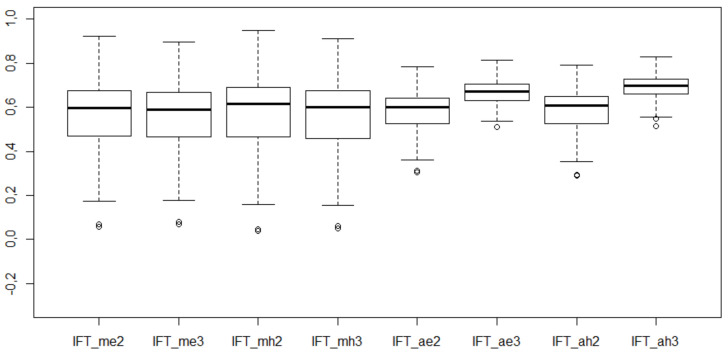
Box plots for IFT values.

Descriptive statistics and box plots for the values of IFT coefficients are presented in Table 14 and Figure 3.

Determining the coordinates of the pattern object based on the values (max, min) resulted in the average IFT values presenting a very similar level with a highly corresponding variability range. In this case, choosing the distance measure and taking into account the degree of uncertainty in its measurement are of no great importance.

Establishing the coordinates of the pattern object based on the intuitionistic values (1,0) significantly reduced the variability range of the IFT values. Moreover, for this type of pattern object, the IFT has become more sensitive to the number of parameters included in measuring the distance between cities and the pattern object. It is evident that the average IFT values increased after taking into account the degree of uncertainty for both the Euclidean and Hamming distances. The variability range of IFT values, in this case, is also the smallest among all the analyzed IFT variants.

The Spearman coefficients between IFT measures are presented in Table 15.

The total consistency of the city rankings using the IFT (Spearman coefficient value equal to 1) was obtained for the Hamming distance with two parameters and for the coordinates of pattern objects determined based on the values (max, min) and (1,0), respectively. However, the lowest still very high consistency of rankings was found in two cases. In the first one, the IFT values were calculated by defining the pattern object coordinates based on the value (1,0) and using the Hamming distance for 2 and 3 parameters, respectively. The second case was very similar and the difference was only in the method used for determining the pattern object coordinates.

The Information Transfer Measures for the IFTes are presented in Table 16.

All the criteria are very well represented by the IFT. It is not possible to identify the IFT variant following which the information transfer for all the criteria is the highest or the lowest. As with the IFSM, an identical information transfer for each criterion was observed for IFT__mh2_ and IFT__ah2_. Regardless of the IFT variant, C_3_ was the least represented, whereas C_1_ received the strongest representation of all the criteria applied.

Spearman coefficients between the IFSM and the IFT measures are presented in Table 17.

The compared measures, regardless of the distance used and the coordinates of the pattern object, rank cities in a very similar way in terms of satisfaction with public administration services. The lowest value of the Spearman coefficient was 0.971, which means very high consistency of all the obtained rankings. In the case of using the pattern object, the coordinates of which were determined based on intuitionistic values (1,0), and the Hamming distance, it did not matter whether the IFSM or the IFT measure was used in ranking the cities (Spearman coefficient value was 1). In this case, it was also irrelevant to include the uncertainty parameter in the calculation of the distance between the cities and the pattern city.

Identical results of the city rankings using the IFSM, and the IFT were also recorded for the combination of the Hamming measure with two parameters and the coordinates of the pattern objects determined based on max and min values. Taking into account the uncertainty parameter in this case resulted in slight changes in the ranking position of some cities.

## 5. Conclusions

The paper proposes the IFSM as a method for measuring complex phenomena based on survey data. Most frequently, this type of data takes the form of ordinal data. In this case the measurement results at the level of individual respondents are not required. The suggested method allows measuring complex phenomena from aggregated ordinal data offered by public statistics. The proposed approach adopts the transformation of aggregated ordinal data into intuitionistic fuzzy sets. The IFSM construction, as with other synthetic measures, requires the researcher to make subjective decisions regarding, e.g., the choice of the distance measure and how to determine the coordinates of the pattern object. Therefore, this paper provides a comparative analysis addressing the two most popular distances for the intuitionistic fuzzy sets, the Euclidean distance and the Hamming distance. Both two and three parameters of the intuitionistic fuzzy sets were taken into account in the distance calculation. The construction of a pattern object based on the intuitionistic values was also proposed and compared with the classical approach, where the coordinates of the pattern object are determined based on the maximum and minimum criterion assessments observed in the research sample. In addition, the findings collected using the IFSM were compared with the IFT since both methods are very similar in their construction and use the idea of pattern (reference) objects.

The empirical example presented in the paper as well as the comparative analyses carried out for different variants of the IFSM method allowed formulating the following conclusions:

- in each of the eight analyzed variants of the synthetic measure construction, the mean values of IFT for the cities were higher than in the case of IFSM. Furthermore, in each of these cases the variability range of IFT values was lower than that of IFSM. This is primarily true when the coordinates of the pattern objects were established based on the intuitionistic values (1,0);
- in the case of the pattern object coordinates determined based on the values (max, min), very similar changes in the ranges of their value variability were observed for the IFSM and IFT, depending on the selected distance measure and the number of parameters included in it;
- determining the coordinates of the pattern objects based on the value (1,0) caused that the values of IFSM and IFT changed in an opposite way as a result of the applied distance and taking into account the degree of uncertainty. The increase in the value of the IFT measure for the cities occurred along with the decrease in the value of IFSM and vice versa. It should be noted, however, that the increase in IFT values took place at a reduced variability range. In the case of IFSM such a large reduction in variability was not observed. Therefore, the application of the IFSM in the variant with the pattern object, the coordinates of which are determined based on the intuitionistic values (1,0), allowed for differentiating cities to a greater extent in terms of the complex phenomenon, i.e., satisfaction with public administration services;
- for the analyzed data set, the ranking of cities determined on the basis of both IFSM and IFT values turned out to be a little sensitive to the choice of the distance measure and the method for determining the coordinates of pattern objects. The values of the correlation coefficients for the obtained rankings were very high, reaching the value of 1 in some cases. Slightly greater consistency of the rankings was obtained for the IFT, which suggests a somewhat higher sensitivity of the IFSM to the choice of the distance measure and the method for determining the coordinates of the pattern object. In the case of both methods, the highest ranking consistency was recorded using the Hamming distance for two parameters and the coordinates of pattern objects established based on the values (max, min) and (1,0), respectively. Therefore, including the third parameter in measuring the distance, taking the form of the degree of uncertainty, changes the position of cities in the rankings, although in the presented example these changes were small and referred to some cities only. The least consistent rankings for both measures were also observed for the Hamming distance, however, for a different number of parameters combined with:
  (a) pattern objects, the coordinates of which were determined based on the values (1,0);
  (b) pattern objects, the coordinates of which were determined based on the values (1,0) and (max, min).
  It should be highlighted that, despite the high consistency of the obtained rankings, the values of measures for cities were diversified, which suggests a different level of residents’ satisfaction with public administration services. It is of particular importance in the context of monitoring the analyzed phenomenon over time because the same position of a city in the ranking does not imply that the level of the phenomenon is not going to increase over time;
- it is difficult to identify, from among the IFSM and the IFT, a better method in terms of representing the particular criteria. All criteria are very well represented by both the IFSM and the IFT. In either case, the largest transfer of information was recorded for C_1_ and the smallest for C_3_.

A certain limitation of the proposed method for transforming ordinal data is that there is no possibility to differentiate categories on the side of “positive” and “negative” responses. This may have an impact on the synthetic measure values and the ranking positions of the assessed objects. One of the directions for further research on the IFSM will be presenting some proposals in this area. The influence of data distribution on the results of object ranking using the IFSM will also be analyzed.

## Figures and Tables

**Table 1:** Classification of variants IFSM methods based on an intuitionistic fuzzy framework with respect to pattern objects and distance measures.

Methods	Pattern Objects	The Distance Measure	Number of Parameters in the Distance Measure
IFSM__me2_	based on max and min values	Euclidean distance	distance based on two parameters
IFSM__me3_	based on max and min values	Euclidean distance	distance based on three parameters
IFSM__mh2_	based on max and min values	Hamming distance	distance based on two parameters
IFSM__mh3_	based on max and min values	Hamming distance	distance based on three parameters
IFSM__ae2_	based on (1,0) values	Euclidean distance	distance based on two parameters
IFSM__ae3_	based on (1,0) values	Euclidean distance	distance based on three parameters
IFSM__ah2_	based on (1,0) values	Hamming distance	distance based on two parameters
IFSM__ah3_	based on (1,0) values	Hamming distance	distance based on three parameters

**Table 2:** Sociodemographic characteristics of the respondents.

Feature	Category	Percentage
Gender	Male	47.739%
	Female	52.261%
Age	15–19	4.965%
	20–24	9.288%
	25–34	18.683%
	35–44	17.592%
	45–54	15.872%
	55–64	13.920%
	65–74	11.407%
	75+	8.273%
Education	Less than Primary education	0.173%
	Primary education	1.308%
	Lower secondary education	10.389%
	Upper secondary education	35.257%
	Post-secondary non-tertiary education	8.056%
	Short-cycle tertiary education	12.886%
	Bachelor or equivalent	18.269%
	Master or equivalent	10.986%
	Doctoral or equivalent	2.221%
	Don’t know/No Answer/Refuses	0.455%

Source: [33].

**Table 3:** The assessment of cities.

City	Category *	C_1_	C_2_	C_3_	C_4_	C_5_
Palermo	1	45.85%	28.70%	36.42%	15.81%	6.24%
	2	37.74%	40.18%	40.07%	29.24%	14.32%
	3	10.94%	23.62%	19.53%	42.20%	41.56%
	4	1.88%	4.41%	2.55%	8.79%	30.36%
	99	3.59%	3.10%	1.43%	3.96%	7.52%
Zurich	1	1.70%	2.68%	1.90%	0.49%	33.15%
	2	10.67%	17.88%	16.88%	7.82%	34.05%
	3	45.59%	46.67%	51.07%	33.17%	14.56%
	4	27.74%	25.63%	26.04%	46.91%	2.58%
	99	14.30%	7.13%	4.11%	11.61%	15.65%
	Total	700	700	700	700	700

* 1—Strongly disagree, 2—Somewhat disagree, 3—Somewhat agree, 4—Strongly agree, 99—Don’t know/No Answer/Refuses. Source: [33].

**Table 4:** The assessment of cities using the IFVs.

City	Parameter	C_1_	C_2_	C_3_	C_4_	C_5_
Zurich	ν	0.124	0.206	0.188	0.083	0.171
	μ	0.733	0.723	0.771	0.801	0.672
	π	0.143	0.071	0.041	0.116	0.157
Palermo	ν	0.836	0.689	0.765	0.451	0.719
	μ	0.128	0.280	0.221	0.510	0.206
	π	0.036	0.031	0.014	0.040	0.075

Source: [33].

**Table 5:** The coordinates of an intuitionistic fuzzy pattern object based on (1,0) values.

Parameter	C_1_	C_2_	C_3_	C_4_	C_5_
ν	0	0	0	0	0
μ	1	1	1	1	1
π	0	0	0	0	0

**Table 6:** The coordinates of an intuitionistic fuzzy pattern object based on max and min values.

Parameter	C_1_	C_2_	C_3_	C_4_	C_5_
ν	0.124	0.199	0.147	0.080	0.164
μ	0.733	0.789	0.771	0.846	0.789
π	0.143	0.013	0.082	0.074	0.047

**Table 7:** Distances and IFSM values.

City	Measure	IFSM__me2_	IFSM__me3_	IFSM__mh2_	IFSM__mh3_	IFSM__ae2_	IFSM__ae3_	IFSM__ah2_	IFSM__ah3_
Zurich	$d^{+}$	0.047	0.064	0.029	0.054	0.218	0.233	0.207	0.260
	$d_{0}$	0.460	0.465	0.439	0.474	0.638	0.643	0.618	0.662
	IFSM _value_	0.899	0.863	0.935	0.887	0.658	0.638	0.665	0.607
Palermo	$d^{+}$	0.544	0.545	0.533	0.558	0.724	0.724	0.711	0.731
	$d_{0}$	0.460	0.465	0.439	0.474	0.638	0.643	0.618	0.662
	IFSM _value_	−0.183	−0.177	−0.214	−0.178	−0.135	−0.127	−0.152	−0.105

**Table 8:** Descriptive statistics for IFSM values.

Descriptive Statistics	Satisfaction with Administration							
	IFSM__me2_	IFSM__me3_	IFSM__mh2_	IFSM__mh3_	IFSM__ae2_	IFSM__ae3_	IFSM__ah2_	IFSM__ah3_
Min	−0.183	−0.177	−0.214	−0.178	−0.135	−0.127	−0.152	−0.105
Max	0.899	0.863	0.935	0.887	0.658	0.638	0.665	0.607
Range	1.081	1.039	1.149	1.064	0.792	0.765	0.817	0.712
Average	0.444	0.426	0.469	0.433	0.325	0.313	0.334	0.298
Standard deviation	0.444	0.426	0.469	0.433	0.325	0.313	0.334	0.298

**Table 9:** Spearman coefficients between IFSM measures.

Coefficient	IFSM__me2_	IFSM__me3_	IFSM__mh2_	IFSM__mh3_	IFSM__ae2_	IFSM__ae3_	IFSM__ah2_	IFSM__ah3_
IFSM__me2_	1.000	0.958 **	0.927 **	0.920 **	0.971 **	0.949 **	0.927 **	0.872 **
IFSM__me3_		1.000	0.898 **	0.922 **	0.939 **	0.952 **	0.898 **	0.884 **
IFSM__mh2_			1.000	0.929 **	0.945 **	0.917 **	1.000 **	0.870 **
IFSM__mh3_				1.000	0.926 **	0.936 **	0.929 **	0.911 **
IFSM__ae2_					1.000	0.958 **	0.945 **	0.887 **
IFSM__ae3_						1.000	0.917 **	0.921 **
IFSM__ah2_							1.000	0.870 **
IFSM__ah3_								1.000
IFSM differ with distance measure parameters (2 or 3)		IFSM differ with distance measures (Hamming or Euclidean)		IFSM differ with pattern (based on (1,0) or based on max, min values)		IFSM differ with all elements: distance measures parameters, distance measure function, and pattern		

** *p* = 0.01.

**Table 10:** The Information Transfer Measures for IFSMes.

Criteria	IFSM__me2_	IFSM__me3_	IFSM__mh2_	IFSM__mh3_	IFSM__ae2_	IFSM__ae3_	IFSM__ah2_	IFSM__ah3_
C_1_	0.879 **	0.862 **	0.906 **	0.881 **	0.891 **	0.716 **	0.906 **	0.870 **
C_2_	0.762 **	0.764 **	0.781 **	0.786 **	0.781 **	0.593 **	0.781 **	0.803 **
C_3_	0.668 **	0.650 **	0.716 **	0.691 **	0.679 **	0.503 **	0.716 **	0.649 **
C_4_	0.739 **	0.739 **	0.764 **	0.766 **	0.733 **	0.556 **	0.764 **	0.750 **
C_5_	0.858 **	0.860 **	0.816 **	0.816 **	0.845 **	0.647 **	0.816 **	0.795 **

** *p* = 0.01.

**Table 11:** A classification of variants of the IFTes with respect to pattern objects, and distance measure.

Methods	Pattern Objects	The Distance Measure	Number of Parameters in the Distance Measure
IFT__me2_	based on max and min values	Euclidean distance	distance based on two parameters
IFT__me3_	based on max and min values	Euclidean distance	distance based on three parameters
IFT__mh2_	based on max and min values	Hamming distance	distance based on two parameters
IFT__mh3_	based on max and min values	Hamming distance	distance based on three parameters
IFT__ae2_	based on (1,0) and (0,1) values	Euclidean distance	distance based on two parameters
IFT__ae3_	based on (1,0) and (0,1) values	Euclidean distance	distance based on three parameters
IFT__ah2_	based on (1,0) and (0,1) values	Hamming distance	distance based on two parameters
IFT__ah3_	based on (1,0) and (0,1) values	Hamming distance	distance based on three parameters

**Table 12:** The coordinates of an intuitionistic fuzzy anti-pattern object based on (0,1) values (used in IFT method).

Parameter	C_1_	C_2_	C_3_	C_4_	C_5_
ν	1	1	1	1	1
μ	0	0	0	0	0
π	0	0	0	0	0

**Table 13:** The coordinates of an intuitionistic fuzzy anti-pattern object based on max and min values (used in IFT method).

Parameter	C_1_	C_2_	C_3_	C_4_	C_5_
ν	0.836	0.710	0.765	0.450	0.772
μ	0.128	0.271	0.221	0.501	0.088
π	0.036	0.019	0.014	0.048	0.140

**Table 14:** Descriptive statistics for IFT values.

Descriptive Statistics	Satisfaction with Administration							
	IFT__me2_	IFT__me3_	IFT__mh2_	IFT__mh3_	IFT__ae2_	IFT__ae3_	IFT__ah2_	IFT__ah3_
Min	0.058	0.070	0.038	0.050	0.305	0.509	0.289	0.513
Max	0.920	0.895	0.948	0.911	0.785	0.814	0.793	0.827
Range	0.862	0.824	0.910	0.861	0.480	0.305	0.504	0.314
Average	0.570	0.563	0.579	0.565	0.584	0.668	0.588	0.690
Standard deviation	0.173	0.166	0.186	0.173	0.097	0.064	0.103	0.066

**Table 15:** Spearman coefficients between IFTes.

Coefficient	IFT__me2_	IFT__me3_	IFT__mh2_	IFT__mh3_	IFT__ae2_	IFT__ae3_	IFT__ah2_	IFT__ah3_
IFT__me2_	1.000	0.996 **	0.994 **	0.998 **	0.986 **	0.996 **	0.972 **	0.999 **
IFT__me3_		1.000	0.995 **	0.994 **	0.997 **	0.987 **	0.995 **	0.972 **
IFT__mh2_			1.000	0.997 **	0.999 **	0.983 **	1.000 **	0.971 **
IFT__mh3_				1.000	0.995 **	0.978 **	0.997 **	0.911 **
IFT__ae2_					1.000	0.987 **	0.999 **	0.975 **
IFT__ae3_						1.000	0.983 **	0.995 **
IFT__ah2_							1.000	0.971 **
IFT__ah3_								1.000
IFT measures differ with distance measure parameters (2 or 3)		IFT measures differ with distance measures (Hamming or Euclidean)		IFT measures differ with pattern (based on (1,0) or based on max, min values)		IFT measures differ with all elements: distance measures parameters, distance measure function, and pattern		

** *p* = 0.01.

**Table 16:** The Information Transfer Measures for the IFTes.

Criteria	IFT__me2_	IFT__me3_	IFT__mh2_	IFT__mh3_	IFT__ae2_	IFT__ae3_	IFT__ah2_	IFT__ah3_
C_1_	0.896 **	0.891 **	0.906 **	0.896 **	0.904 **	0.875 **	0.906 **	0.870 **
C_2_	0.763 **	0.764 **	0.781 **	0.769 **	0.779 **	0.800 **	0.781 **	0.803 **
C_3_	0.702 **	0.705 **	0.716 **	0.726 **	0.702 **	0.654 **	0.716 **	0.649 **
C_4_	0.742 **	0.741 **	0.764 **	0.766 **	0.756 **	0.740 **	0.764 **	0.750 **
C_5_	0.848 **	0.846 **	0.816 **	0.818 **	0.828 **	0.823 **	0.816 **	0.795 **

** *p* = 0.01.

**Table 17:** Spearman coefficients between the IFSMes and the IFTes.

Coefficient	IFT__me2_	IFT__me3_	IFT__mh2_	IFT__mh3_	IFT__ae2_	IFT__ae3_	IFT__ah2_	IFT__ah3_
IFSM__me2_	0.995 **	0.995 **	0.988 **	0.985 **	0.993 **	0.988 **	0.988 **	0.972 **
IFSM__me3_	0.990 **	0.991 **	0.981 **	0.979 **	0.986 **	0.991 **	0.981 **	0.977 **
IFSM__mh2_	0.996 **	0.995 **	1.000 **	0.997 **	0.999 **	0.983 **	1.000 **	0.971 **
IFSM__mh3_	0.989 **	0.991 **	0.990 **	0.991 **	0.991 **	0.993 **	0.990 **	0.986 **
IFSM__ae2_	0.997 **	0.997 **	0.993 **	0.989 **	0.997 **	0.991 **	0.993 **	0.977 **
IFSM__ae3_	0.992 **	0.993 **	0.987 **	0.983 **	0.992 **	0.997 **	0.987 **	0.988 **
IFSM__ah2_	0.996 **	0.995 **	1.000 **	0.997 **	0.999 **	0.983 **	1.000 **	0.971 **
IFSM__ah3_	0.972 **	0.972 **	0.971 **	0.965 **	0.975 **	0.995 **	0.971 **	1.000 **

** *p* = 0.01.

## Data Availability

Not applicable (for secondary data analysis, see [33]).

## Appendix A

**Table A1:** Degrees of non-membership to IFVs for cities.

City	C_1_	C_2_	C_3_	C_4_	C_5_
Aalborg	0.164	0.293	0.246	0.101	0.166
Amsterdam	0.327	0.367	0.413	0.127	0.281
Ankara	0.409	0.335	0.425	0.242	0.422
Antalya	0.349	0.213	0.344	0.134	0.337
Antwerpen	0.260	0.199	0.371	0.358	0.264
Athina	0.619	0.580	0.724	0.352	0.584
Barcelona	0.540	0.353	0.582	0.302	0.435
Belfast	0.307	0.302	0.323	0.156	0.370
Beograd	0.624	0.617	0.558	0.280	0.749
Berlin	0.579	0.600	0.293	0.269	0.371
Białystok	0.290	0.348	0.292	0.127	0.301
Bologna	0.442	0.459	0.487	0.192	0.386
Bordeaux	0.382	0.357	0.321	0.217	0.265
Braga	0.456	0.303	0.402	0.250	0.535
Bratislava	0.333	0.465	0.267	0.247	0.498
Bruxelles	0.363	0.211	0.344	0.248	0.284
Bucharest	0.458	0.490	0.321	0.280	0.639
Budapest	0.395	0.337	0.376	0.131	0.373
Burgas	0.439	0.392	0.476	0.141	0.535
Cardiff	0.238	0.269	0.295	0.139	0.196
Cluj-Napoca	0.279	0.316	0.249	0.179	0.580
Diyarbakir	0.572	0.494	0.381	0.394	0.507
Dortmund	0.460	0.527	0.450	0.248	0.396
Dublin	0.284	0.249	0.264	0.132	0.339
Essen	0.368	0.530	0.323	0.212	0.236
Gdańsk	0.316	0.344	0.280	0.155	0.356
Genève	0.159	0.200	0.362	0.232	0.384
Glasgow	0.381	0.318	0.368	0.172	0.330
Graz	0.316	0.254	0.277	0.108	0.225
Groningen	0.188	0.199	0.407	0.080	0.167
Hamburg	0.302	0.449	0.300	0.196	0.251
Helsinki	0.373	0.505	0.297	0.218	0.341
Irakleio	0.651	0.548	0.716	0.193	0.627
Istanbul	0.463	0.331	0.428	0.275	0.564
København	0.299	0.323	0.206	0.100	0.167
Košice	0.346	0.357	0.284	0.173	0.481
Kraków	0.311	0.479	0.393	0.161	0.298
Lefkosia	0.461	0.206	0.425	0.135	0.593
Leipzig	0.256	0.329	0.367	0.157	0.203
Liège	0.309	0.220	0.360	0.228	0.312
Lille	0.434	0.332	0.385	0.279	0.255
Lisboa	0.574	0.476	0.501	0.262	0.572
Ljubljana	0.347	0.323	0.207	0.175	0.563
London	0.374	0.340	0.342	0.144	0.259
Luxembourg	0.262	0.289	0.209	0.147	0.310
Madrid	0.418	0.382	0.446	0.264	0.398
Málaga	0.402	0.335	0.386	0.229	0.417
Malmö	0.334	0.464	0.235	0.173	0.252
Manchester	0.243	0.261	0.272	0.115	0.387
Marseille	0.429	0.397	0.503	0.361	0.457
Miskolc	0.232	0.271	0.362	0.083	0.439
Munich	0.354	0.401	0.273	0.156	0.191
Naples	0.719	0.626	0.711	0.411	0.607
Oslo	0.452	0.460	0.330	0.222	0.279
Ostrava	0.363	0.436	0.266	0.123	0.534
Oulu	0.349	0.455	0.372	0.240	0.286
Oviedo	0.466	0.444	0.527	0.301	0.472
Palermo	0.836	0.689	0.765	0.450	0.719
Paris	0.423	0.379	0.401	0.256	0.305
Piatra Neamt	0.327	0.360	0.283	0.194	0.562
Podgorica	0.515	0.427	0.317	0.310	0.670
Praha	0.396	0.439	0.190	0.149	0.471
Rennes	0.368	0.311	0.315	0.201	0.164
Reykjavík	0.516	0.496	0.506	0.211	0.570
Riga	0.443	0.471	0.712	0.268	0.681
Rome	0.827	0.710	0.727	0.402	0.772
Rostock	0.199	0.386	0.254	0.175	0.234
Rotterdam	0.380	0.334	0.311	0.185	0.223
Skopje	0.675	0.437	0.423	0.328	0.764
Sofia	0.524	0.520	0.461	0.248	0.503
Stockholm	0.368	0.427	0.217	0.174	0.225
Strasbourg	0.322	0.304	0.359	0.246	0.252
Tallinn	0.251	0.313	0.147	0.127	0.563
Tirana	0.482	0.396	0.510	0.229	0.756
Turin	0.642	0.611	0.648	0.299	0.492
Tyneside conurbation	0.280	0.263	0.331	0.177	0.270
Valletta	0.319	0.252	0.200	0.141	0.185
Verona	0.530	0.541	0.395	0.235	0.596
Vilnius	0.393	0.342	0.385	0.251	0.423
Warszawa	0.391	0.485	0.435	0.191	0.331
Wien	0.232	0.310	0.266	0.119	0.226
Zagreb	0.654	0.601	0.588	0.220	0.753
Zurich	0.124	0.206	0.188	0.083	0.171

**Table A2:** Degrees of membership to IFVs for cities.

City	C_1_	C_2_	C_3_	C_4_	C_5_
Aalborg	0.670	0.608	0.568	0.846	0.788
Amsterdam	0.487	0.571	0.524	0.805	0.447
Ankara	0.562	0.642	0.562	0.725	0.492
Antalya	0.644	0.780	0.639	0.817	0.518
Antwerpen	0.557	0.724	0.619	0.526	0.468
Athina	0.374	0.405	0.264	0.526	0.174
Barcelona	0.446	0.628	0.408	0.668	0.481
Belfast	0.500	0.630	0.581	0.690	0.418
Beograd	0.328	0.359	0.405	0.596	0.088
Berlin	0.326	0.315	0.602	0.553	0.293
Białystok	0.687	0.612	0.678	0.764	0.442
Bologna	0.518	0.508	0.500	0.744	0.485
Bordeaux	0.575	0.587	0.598	0.724	0.484
Braga	0.485	0.660	0.564	0.677	0.281
Bratislava	0.509	0.462	0.659	0.667	0.231
Bruxelles	0.627	0.789	0.619	0.700	0.498
Bucharest	0.458	0.491	0.637	0.552	0.118
Budapest	0.459	0.567	0.523	0.677	0.304
Burgas	0.519	0.567	0.480	0.802	0.305
Cardiff	0.572	0.651	0.637	0.754	0.573
Cluj-Napoca	0.628	0.601	0.696	0.630	0.131
Diyarbakir	0.375	0.495	0.599	0.574	0.428
Dortmund	0.504	0.435	0.479	0.654	0.341
Dublin	0.589	0.659	0.637	0.777	0.483
Essen	0.566	0.364	0.574	0.658	0.382
Gdańsk	0.632	0.613	0.683	0.781	0.378
Genève	0.717	0.746	0.573	0.684	0.384
Glasgow	0.477	0.558	0.529	0.669	0.492
Graz	0.628	0.711	0.697	0.831	0.591
Groningen	0.551	0.607	0.543	0.827	0.600
Hamburg	0.629	0.476	0.579	0.714	0.418
Helsinki	0.391	0.398	0.609	0.723	0.598
Irakleio	0.324	0.424	0.265	0.656	0.315
Istanbul	0.508	0.630	0.517	0.681	0.323
København	0.589	0.580	0.616	0.833	0.789
Košice	0.569	0.579	0.674	0.732	0.228
Kraków	0.593	0.478	0.564	0.746	0.369
Lefkosia	0.531	0.771	0.534	0.736	0.362
Leipzig	0.631	0.596	0.551	0.634	0.378
Liège	0.569	0.747	0.613	0.606	0.396
Lille	0.520	0.637	0.551	0.615	0.432
Lisboa	0.340	0.492	0.460	0.647	0.238
Ljubljana	0.544	0.597	0.675	0.699	0.235
London	0.496	0.585	0.579	0.764	0.519
Luxembourg	0.681	0.690	0.764	0.838	0.612
Madrid	0.540	0.574	0.508	0.632	0.412
Málaga	0.502	0.585	0.517	0.660	0.407
Malmö	0.507	0.438	0.574	0.748	0.672
Manchester	0.611	0.695	0.642	0.754	0.537
Marseille	0.500	0.549	0.427	0.569	0.248
Miskolc	0.511	0.582	0.566	0.706	0.271
Munich	0.500	0.516	0.644	0.731	0.488
Naples	0.246	0.354	0.270	0.501	0.250
Oslo	0.291	0.333	0.491	0.640	0.548
Ostrava	0.478	0.480	0.674	0.810	0.248
Oulu	0.549	0.468	0.561	0.688	0.605
Oviedo	0.471	0.511	0.424	0.595	0.379
Palermo	0.128	0.280	0.221	0.510	0.206
Paris	0.544	0.597	0.532	0.709	0.445
Piatra Neamt	0.594	0.563	0.682	0.540	0.179
Podgorica	0.437	0.524	0.613	0.626	0.146
Praha	0.415	0.416	0.688	0.718	0.241
Rennes	0.616	0.654	0.648	0.747	0.624
Reykjavík	0.273	0.410	0.453	0.643	0.353
Riga	0.414	0.483	0.252	0.634	0.205
Rome	0.155	0.271	0.261	0.522	0.156
Rostock	0.649	0.527	0.698	0.693	0.449
Rotterdam	0.509	0.611	0.629	0.723	0.420
Skopje	0.315	0.531	0.551	0.584	0.108
Sofia	0.323	0.370	0.523	0.608	0.175
Stockholm	0.426	0.450	0.624	0.675	0.538
Strasbourg	0.644	0.670	0.594	0.670	0.490
Tallinn	0.535	0.547	0.631	0.793	0.282
Tirana	0.485	0.588	0.474	0.715	0.220
Turin	0.299	0.361	0.332	0.558	0.362
Tyneside conurbation	0.540	0.669	0.593	0.620	0.488
Valletta	0.542	0.652	0.617	0.723	0.489
Verona	0.411	0.423	0.568	0.655	0.281
Vilnius	0.452	0.561	0.496	0.679	0.359
Warszawa	0.536	0.454	0.520	0.779	0.363
Wien	0.678	0.669	0.707	0.819	0.615
Zagreb	0.313	0.340	0.377	0.557	0.092
Zurich	0.733	0.723	0.771	0.801	0.672

**Table A3:** Degrees of hesitancy for IFVs for cities.

City	C_1_	C_2_	C_3_	C_4_	C_5_
Aalborg	0.166	0.099	0.186	0.053	0.045
Amsterdam	0.186	0.062	0.063	0.067	0.272
Ankara	0.029	0.023	0.013	0.033	0.086
Antalya	0.007	0.007	0.017	0.050	0.145
Antwerpen	0.183	0.077	0.010	0.116	0.267
Athina	0.007	0.015	0.012	0.122	0.242
Barcelona	0.014	0.019	0.009	0.030	0.084
Belfast	0.192	0.068	0.096	0.154	0.211
Beograd	0.048	0.024	0.037	0.124	0.163
Berlin	0.095	0.085	0.104	0.178	0.336
Białystok	0.023	0.040	0.030	0.109	0.256
Bologna	0.039	0.033	0.013	0.064	0.130
Bordeaux	0.043	0.056	0.081	0.059	0.252
Braga	0.059	0.037	0.034	0.073	0.184
Bratislava	0.158	0.073	0.074	0.085	0.271
Bruxelles	0.010	0.000	0.037	0.052	0.218
Bucharest	0.084	0.019	0.042	0.169	0.243
Budapest	0.146	0.096	0.101	0.192	0.322
Burgas	0.042	0.041	0.044	0.056	0.160
Cardiff	0.190	0.079	0.068	0.107	0.231
Cluj-Napoca	0.093	0.082	0.055	0.192	0.289
Diyarbakir	0.053	0.011	0.020	0.032	0.065
Dortmund	0.036	0.037	0.070	0.099	0.263
Dublin	0.127	0.092	0.099	0.091	0.178
Essen	0.066	0.106	0.103	0.130	0.382
Gdańsk	0.052	0.043	0.037	0.063	0.266
Genève	0.124	0.054	0.065	0.084	0.232
Glasgow	0.142	0.124	0.103	0.159	0.178
Graz	0.056	0.035	0.025	0.061	0.183
Groningen	0.261	0.194	0.050	0.093	0.233
Hamburg	0.069	0.075	0.120	0.090	0.331
Helsinki	0.236	0.097	0.094	0.059	0.061
Irakleio	0.026	0.028	0.019	0.152	0.058
Istanbul	0.029	0.039	0.055	0.044	0.113
København	0.112	0.096	0.177	0.068	0.044
Košice	0.084	0.064	0.042	0.096	0.291
Kraków	0.097	0.043	0.043	0.094	0.333
Lefkosia	0.008	0.023	0.041	0.129	0.045
Leipzig	0.113	0.074	0.082	0.209	0.419
Liège	0.121	0.033	0.027	0.166	0.292
Lille	0.047	0.031	0.064	0.106	0.313
Lisboa	0.086	0.031	0.039	0.091	0.190
Ljubljana	0.109	0.080	0.118	0.126	0.202
London	0.130	0.075	0.079	0.091	0.222
Luxembourg	0.057	0.021	0.027	0.015	0.078
Madrid	0.042	0.044	0.046	0.105	0.190
Málaga	0.095	0.080	0.096	0.111	0.176
Malmö	0.159	0.098	0.190	0.079	0.076
Manchester	0.146	0.045	0.086	0.132	0.076
Marseille	0.070	0.053	0.070	0.070	0.295
Miskolc	0.257	0.147	0.072	0.210	0.290
Munich	0.146	0.082	0.083	0.112	0.321
Naples	0.036	0.020	0.020	0.088	0.142
Oslo	0.257	0.207	0.179	0.138	0.174
Ostrava	0.158	0.085	0.060	0.068	0.218
Oulu	0.102	0.078	0.068	0.072	0.109
Oviedo	0.062	0.046	0.049	0.104	0.149
Palermo	0.036	0.031	0.014	0.040	0.075
Paris	0.033	0.023	0.067	0.034	0.250
Piatra Neamt	0.079	0.077	0.035	0.266	0.259
Podgorica	0.048	0.050	0.070	0.063	0.184
Praha	0.190	0.145	0.121	0.133	0.288
Rennes	0.016	0.035	0.037	0.052	0.212
Reykjavík	0.211	0.094	0.041	0.147	0.077
Riga	0.144	0.046	0.036	0.098	0.114
Rome	0.018	0.019	0.012	0.076	0.072
Rostock	0.153	0.087	0.047	0.132	0.317
Rotterdam	0.111	0.055	0.060	0.092	0.357
Skopje	0.010	0.032	0.025	0.089	0.129
Sofia	0.153	0.111	0.016	0.144	0.322
Stockholm	0.206	0.123	0.158	0.151	0.237
Strasbourg	0.035	0.026	0.047	0.084	0.259
Tallinn	0.215	0.141	0.222	0.080	0.155
Tirana	0.034	0.015	0.015	0.055	0.024
Turin	0.059	0.028	0.020	0.143	0.145
Tyneside conurbation	0.180	0.068	0.076	0.204	0.242
Valletta	0.139	0.096	0.183	0.136	0.326
Verona	0.059	0.036	0.037	0.110	0.123
Vilnius	0.154	0.098	0.118	0.070	0.218
Warszawa	0.074	0.061	0.046	0.030	0.306
Wien	0.090	0.021	0.027	0.062	0.159
Zagreb	0.034	0.059	0.034	0.223	0.154
Zurich	0.143	0.071	0.041	0.116	0.157

**Table A4:** Values of IFSM coefficients for cities.

City	IFSM__me2_	IFSM__me3_	IFSM__mh2_	IFSM__mh3_	IFSM__ae2_	IFSM__ae3_	IFSM__ah2_	IFSM__ah3_
Aalborg	0.784	0.766	0.839	0.801	0.577	0.558	0.597	0.541
Amsterdam	0.542	0.518	0.568	0.528	0.388	0.369	0.404	0.346
Ankara	0.529	0.523	0.529	0.507	0.384	0.387	0.376	0.390
Antalya	0.675	0.655	0.728	0.679	0.507	0.505	0.517	0.516
Antwerpen	0.554	0.527	0.596	0.534	0.420	0.399	0.424	0.364
Athina	0.030	0.023	0.014	−0.008	0.020	0.019	0.010	0.016
Barcelona	0.356	0.352	0.363	0.350	0.263	0.267	0.258	0.284
Belfast	0.565	0.548	0.578	0.533	0.403	0.384	0.411	0.341
Beograd	0.024	0.025	0.028	0.033	0.016	0.018	0.020	0.026
Berlin	0.240	0.216	0.262	0.204	0.174	0.155	0.187	0.120
Białystok	0.649	0.612	0.684	0.614	0.476	0.462	0.486	0.451
Bologna	0.446	0.441	0.448	0.428	0.319	0.320	0.318	0.322
Bordeaux	0.590	0.564	0.592	0.546	0.424	0.412	0.421	0.386
Braga	0.400	0.394	0.432	0.412	0.298	0.296	0.307	0.295
Bratislava	0.392	0.376	0.431	0.407	0.289	0.275	0.307	0.253
Bruxelles	0.637	0.609	0.674	0.617	0.478	0.470	0.479	0.466
Bucharest	0.243	0.233	0.284	0.253	0.185	0.177	0.202	0.171
Budapest	0.449	0.414	0.477	0.411	0.323	0.296	0.339	0.254
Burgas	0.384	0.380	0.425	0.405	0.283	0.283	0.302	0.297
Cardiff	0.723	0.692	0.735	0.682	0.516	0.492	0.522	0.452
Cluj-Napoca	0.399	0.374	0.515	0.444	0.318	0.299	0.366	0.301
Diyarbakir	0.310	0.311	0.296	0.303	0.225	0.229	0.210	0.236
Dortmund	0.349	0.334	0.344	0.311	0.248	0.240	0.244	0.218
Dublin	0.671	0.657	0.695	0.663	0.485	0.472	0.494	0.439
Essen	0.436	0.389	0.467	0.384	0.317	0.290	0.332	0.258
Gdańsk	0.599	0.569	0.641	0.584	0.442	0.429	0.455	0.422
Genève	0.593	0.576	0.670	0.637	0.456	0.442	0.477	0.427
Glasgow	0.532	0.518	0.531	0.492	0.377	0.362	0.377	0.312
Graz	0.758	0.733	0.787	0.736	0.553	0.545	0.559	0.534
Groningen	0.673	0.621	0.743	0.649	0.497	0.461	0.528	0.434
Hamburg	0.544	0.503	0.568	0.500	0.394	0.370	0.404	0.340
Helsinki	0.478	0.475	0.492	0.484	0.340	0.330	0.350	0.311
Irakleio	0.085	0.087	0.097	0.104	0.061	0.065	0.069	0.088
Istanbul	0.387	0.385	0.404	0.393	0.286	0.288	0.287	0.293
København	0.744	0.731	0.794	0.763	0.546	0.533	0.565	0.519
Košice	0.462	0.438	0.528	0.475	0.348	0.334	0.375	0.330
Kraków	0.493	0.458	0.520	0.467	0.356	0.336	0.370	0.320
Lefkosia	0.437	0.432	0.522	0.506	0.340	0.341	0.371	0.376
Leipzig	0.565	0.489	0.605	0.508	0.414	0.370	0.430	0.332
Liège	0.571	0.536	0.610	0.547	0.425	0.403	0.434	0.375
Lille	0.506	0.472	0.511	0.457	0.368	0.351	0.363	0.321
Lisboa	0.219	0.218	0.220	0.219	0.156	0.155	0.157	0.147
Ljubljana	0.446	0.437	0.526	0.489	0.341	0.329	0.374	0.320
London	0.594	0.578	0.606	0.578	0.424	0.410	0.431	0.379
Luxembourg	0.782	0.768	0.807	0.771	0.572	0.573	0.574	0.572
Madrid	0.450	0.441	0.440	0.410	0.323	0.319	0.313	0.295
Málaga	0.476	0.470	0.473	0.450	0.340	0.333	0.336	0.296
Malmö	0.578	0.571	0.605	0.583	0.416	0.403	0.430	0.377
Manchester	0.694	0.693	0.714	0.709	0.501	0.492	0.508	0.468
Marseille	0.304	0.287	0.301	0.273	0.221	0.211	0.214	0.182
Miskolc	0.485	0.442	0.552	0.451	0.357	0.322	0.392	0.285
Munich	0.584	0.544	0.610	0.558	0.420	0.394	0.434	0.359
Naples	−0.031	−0.028	−0.063	−0.044	−0.026	−0.022	−0.045	−0.021
Oslo	0.375	0.351	0.395	0.314	0.265	0.240	0.281	0.185
Ostrava	0.412	0.403	0.488	0.466	0.308	0.298	0.347	0.302
Oulu	0.540	0.539	0.534	0.530	0.385	0.382	0.380	0.357
Oviedo	0.325	0.324	0.307	0.300	0.231	0.230	0.218	0.209
Palermo	−0.183	−0.177	−0.214	−0.178	−0.135	−0.127	−0.152	−0.105
Paris	0.511	0.490	0.510	0.467	0.368	0.360	0.363	0.344
Piatra Neamt	0.385	0.356	0.458	0.376	0.297	0.276	0.325	0.262
Podgorica	0.250	0.247	0.292	0.283	0.192	0.191	0.208	0.198
Praha	0.386	0.360	0.457	0.388	0.287	0.263	0.325	0.238
Rennes	0.693	0.662	0.707	0.649	0.505	0.497	0.503	0.483
Reykjavík	0.234	0.234	0.230	0.225	0.161	0.156	0.163	0.133
Riga	0.117	0.122	0.134	0.163	0.086	0.087	0.096	0.090
Rome	−0.178	−0.173	−0.204	−0.163	−0.132	−0.124	−0.145	−0.098
Rostock	0.632	0.586	0.671	0.601	0.463	0.434	0.477	0.401
Rotterdam	0.576	0.528	0.600	0.540	0.417	0.390	0.426	0.363
Skopje	0.108	0.108	0.145	0.144	0.089	0.092	0.103	0.120
Sofia	0.202	0.182	0.209	0.158	0.142	0.127	0.149	0.093
Stockholm	0.537	0.510	0.564	0.488	0.385	0.358	0.401	0.309
Strasbourg	0.621	0.589	0.629	0.577	0.453	0.440	0.447	0.416
Tallinn	0.482	0.463	0.584	0.519	0.367	0.344	0.415	0.331
Tirana	0.245	0.246	0.293	0.299	0.189	0.194	0.208	0.239
Turin	0.110	0.110	0.090	0.088	0.074	0.075	0.064	0.067
Tyneside conurbation	0.618	0.587	0.630	0.568	0.445	0.418	0.448	0.369
Valletta	0.668	0.607	0.706	0.616	0.486	0.447	0.502	0.402
Verona	0.270	0.271	0.277	0.276	0.194	0.195	0.197	0.196
Vilnius	0.438	0.428	0.439	0.416	0.312	0.300	0.312	0.259
Warszawa	0.434	0.408	0.454	0.398	0.312	0.299	0.323	0.290
Wien	0.786	0.769	0.799	0.764	0.566	0.558	0.568	0.543
Zagreb	0.001	−0.002	0.009	−0.015	0.000	−0.002	0.006	−0.004
Zurich	0.899	0.863	0.935	0.887	0.658	0.638	0.665	0.607

**Table A5:** Rank of IFSM coefficients for cities.

City	IFSM__me2_	IFSM__me3_	IFSM__mh2_	IFSM__mh3_	IFSM__ae2_	IFSM__ae3_	IFSM__ah2_	IFSM__ah3_
Aalborg	3	4	2	2	2	4	2	4
Amsterdam	31	31	31	30	31	33	31	31
Ankara	35	30	37	33	34	28	37	20
Antalya	10	11	9	9	8	7	9	7
Antwerpen	29	29	27	27	25	25	27	27
Athina	78	79	79	79	78	78	79	79
Barcelona	61	60	61	60	61	59	61	54
Belfast	28	24	30	28	29	29	30	33
Beograd	79	78	78	78	79	79	78	78
Berlin	70	72	70	72	70	71	70	72
Białystok	14	13	14	16	15	13	14	12
Bologna	47	43	53	48	48	46	53	38
Bordeaux	22	23	28	25	22	21	28	21
Braga	53	52	56	50	54	53	56	49
Bratislava	55	56	57	53	56	58	57	59
Bruxelles	15	14	15	14	14	12	15	10
Bucharest	69	70	68	69	69	69	68	69
Budapest	45	49	47	51	46	52	47	58
Burgas	59	55	58	54	59	56	58	47
Cardiff	7	8	8	8	7	9	8	11
Cluj-Napoca	54	57	42	47	49	50	42	46
Diyarbakir	64	64	65	63	64	64	65	62
Dortmund	62	62	62	62	62	62	62	63
Dublin	12	10	13	10	13	11	13	13
Essen	50	53	49	58	50	54	49	57
Gdańsk	19	22	18	18	20	19	18	16
Genève	21	20	17	13	17	16	17	15
Glasgow	34	32	36	36	35	34	36	42
Graz	5	5	6	6	5	5	6	5
Groningen	11	12	7	11	11	14	7	14
Hamburg	30	34	32	35	30	31	32	34
Helsinki	41	37	45	39	43	43	45	43
Irakleio	77	77	76	76	77	77	76	76
Istanbul	56	54	59	56	58	55	59	51
København	6	6	5	5	6	6	5	6
Košice	43	45	38	40	41	41	38	37
Kraków	38	41	41	41	40	40	41	40
Lefkosia	49	47	40	34	45	39	40	24
Leipzig	27	36	25	32	28	32	25	35
Liège	26	27	22	24	21	24	22	25
Lille	37	38	43	44	37	37	43	39
Lisboa	72	71	72	71	72	72	72	70
Ljubljana	46	46	39	37	42	44	39	41
London	20	19	23	20	23	22	23	22
Luxembourg	4	3	3	3	3	2	3	2
Madrid	44	44	54	52	47	47	54	50
Málaga	42	39	48	46	44	42	48	48
Malmö	24	21	24	19	27	23	24	23
Manchester	8	7	10	7	10	10	10	9
Marseille	65	65	64	68	65	65	64	68
Miskolc	39	42	34	45	39	45	34	53
Munich	23	25	21	23	24	26	21	29
Naples	81	81	81	81	81	81	81	81
Oslo	60	61	60	61	60	61	60	67
Ostrava	52	51	46	43	53	51	46	45
Oulu	32	26	35	29	32	30	35	30
Oviedo	63	63	63	64	63	63	63	64
Palermo	83	83	83	83	83	83	83	83
Paris	36	35	44	42	36	35	44	32
Piatra Neamt	58	59	50	59	55	57	50	55
Podgorica	67	67	67	66	67	68	67	65
Praha	57	58	51	57	57	60	51	61
Rennes	9	9	11	12	9	8	11	8
Reykjavík	71	69	71	70	71	70	71	71
Riga	74	74	75	73	75	75	75	75
Rome	82	82	82	82	82	82	82	82
Rostock	16	18	16	17	16	18	16	19
Rotterdam	25	28	26	26	26	27	26	28
Skopje	76	76	74	75	74	74	74	73
Sofia	73	73	73	74	73	73	73	74
Stockholm	33	33	33	38	33	36	33	44
Strasbourg	17	16	20	21	18	17	20	17
Tallinn	40	40	29	31	38	38	29	36
Tirana	68	68	66	65	68	67	66	60
Turin	75	75	77	77	76	76	77	77
Tyneside conurbation	18	17	19	22	19	20	19	26
Valletta	13	15	12	15	12	15	12	18
Verona	66	66	69	67	66	66	69	66
Vilnius	48	48	55	49	52	48	55	56
Warszawa	51	50	52	55	51	49	52	52
Wien	2	2	4	4	4	3	4	3
Zagreb	80	80	80	80	80	80	80	80
Zurich	1	1	1	1	1	1	1	1

**Table A6:** Values of Sc function and rank of criteria.

City	Sc_1_	Sc_2_	Sc_3_	Sc_4_	Sc_5_	Rank C_1_	Rank C_2_	Rank C_3_	Rank C_4_	Rank C_5_
Aalborg	0.505	0.315	0.323	0.745	0.622	3	21	25	2	1
Amsterdam	0.159	0.204	0.111	0.678	0.166	38	43	59	10	29
Ankara	0.153	0.307	0.137	0.483	0.069	39	23	54	41	39
Antalya	0.294	0.567	0.295	0.683	0.181	19	2	31	9	25
Antwerpen	0.297	0.525	0.248	0.168	0.204	18	6	39	80	23
Athina	−0.245	−0.175	−0.460	0.174	−0.411	74	76	80	79	73
Barcelona	−0.095	0.275	−0.174	0.366	0.047	67	28	75	64	41
Belfast	0.193	0.329	0.258	0.534	0.048	35	20	36	32	40
Beograd	−0.297	−0.258	−0.153	0.316	−0.661	76	78	74	70	82
Berlin	−0.253	−0.285	0.309	0.284	−0.078	75	81	29	73	50
Białystok	0.398	0.264	0.387	0.637	0.141	7	32	18	15	34
Bologna	0.076	0.049	0.013	0.552	0.099	51	54	67	28	36
Bordeaux	0.194	0.229	0.276	0.507	0.219	34	39	33	36	19
Braga	0.029	0.357	0.161	0.427	−0.253	59	18	52	54	62
Bratislava	0.177	−0.003	0.392	0.420	−0.267	36	64	16	57	63
Bruxelles	0.264	0.577	0.275	0.452	0.214	25	1	34	45	22
Bucharest	0.000	0.002	0.316	0.272	−0.520	64	61	27	74	77
Budapest	0.064	0.231	0.147	0.546	−0.069	54	38	53	29	49
Burgas	0.079	0.176	0.004	0.661	−0.229	50	47	68	12	57
Cardiff	0.334	0.382	0.342	0.615	0.377	12	15	22	19	8
Cluj-Napoca	0.349	0.285	0.447	0.451	−0.449	11	26	6	47	74
Diyarbakir	−0.196	0.000	0.219	0.181	−0.079	70	62	42	78	51
Dortmund	0.045	−0.092	0.029	0.406	−0.055	58	69	66	60	47
Dublin	0.305	0.410	0.373	0.645	0.144	17	10	19	13	33
Essen	0.197	−0.166	0.251	0.445	0.147	32	75	38	49	32
Gdańsk	0.316	0.269	0.403	0.626	0.023	15	30	14	16	43
Genève	0.558	0.546	0.211	0.452	0.000	2	4	43	46	45
Glasgow	0.095	0.240	0.162	0.497	0.161	48	36	50	39	30
Graz	0.312	0.456	0.420	0.723	0.366	16	8	9	4	9
Groningen	0.363	0.409	0.136	0.747	0.432	10	11	55	1	5
Hamburg	0.326	0.026	0.279	0.518	0.167	13	56	32	35	28
Helsinki	0.018	−0.107	0.313	0.505	0.257	61	70	28	37	17
Irakleio	−0.327	−0.124	−0.450	0.463	−0.313	77	72	79	43	66
Istanbul	0.045	0.299	0.089	0.407	−0.240	57	25	62	59	60
København	0.290	0.257	0.410	0.733	0.622	20	33	11	3	2
Košice	0.223	0.221	0.390	0.559	−0.253	29	40	17	27	61
Kraków	0.282	−0.001	0.171	0.585	0.071	22	63	48	22	38
Lefkosia	0.070	0.566	0.110	0.601	−0.231	53	3	61	20	59
Leipzig	0.375	0.267	0.184	0.477	0.175	8	31	46	42	27
Liège	0.260	0.526	0.253	0.379	0.084	27	5	37	62	37
Lille	0.086	0.305	0.165	0.337	0.177	49	24	49	68	26
Lisboa	−0.234	0.016	−0.041	0.385	−0.335	72	58	70	61	70
Ljubljana	0.196	0.274	0.468	0.525	−0.328	33	29	5	33	68
London	0.122	0.245	0.238	0.620	0.260	44	35	40	18	16
Luxembourg	0.419	0.402	0.555	0.691	0.302	6	13	2	7	13
Madrid	0.122	0.193	0.062	0.368	0.013	43	45	64	63	44
Málaga	0.100	0.249	0.131	0.432	−0.010	47	34	57	52	46
Malmö	0.173	−0.027	0.339	0.574	0.419	37	66	23	25	6
Manchester	0.368	0.434	0.370	0.639	0.149	9	9	21	14	31
Marseille	0.071	0.152	−0.076	0.208	−0.209	52	48	72	77	55
Miskolc	0.278	0.310	0.204	0.623	−0.168	23	22	44	17	54
Munich	0.147	0.115	0.371	0.575	0.297	40	50	20	24	14
Naples	−0.473	−0.272	−0.441	0.090	−0.357	81	80	78	82	71
Oslo	−0.162	−0.127	0.162	0.418	0.269	69	73	51	58	15
Ostrava	0.115	0.044	0.407	0.687	−0.286	46	55	12	8	65
Oulu	0.201	0.013	0.189	0.448	0.318	31	59	45	48	10
Oviedo	0.005	0.067	−0.103	0.295	−0.092	62	53	73	72	52
Palermo	−0.708	−0.408	−0.544	0.059	−0.514	83	82	83	83	76
Paris	0.121	0.218	0.131	0.453	0.140	45	42	56	44	35
Piatra Neamt	0.268	0.203	0.399	0.346	−0.382	24	44	15	67	72
Podgorica	−0.078	0.097	0.295	0.316	−0.524	66	51	30	71	78
Praha	0.019	−0.024	0.498	0.568	−0.230	60	65	3	26	58
Rennes	0.248	0.343	0.332	0.545	0.460	28	19	24	30	4
Reykjavík	−0.243	−0.086	−0.052	0.432	−0.217	73	68	71	51	56
Riga	−0.029	0.013	−0.460	0.366	−0.475	65	60	81	65	75
Rome	−0.672	−0.439	−0.466	0.120	−0.616	82	83	82	81	80
Rostock	0.450	0.141	0.444	0.519	0.215	4	49	7	34	21
Rotterdam	0.128	0.277	0.318	0.537	0.196	42	27	26	31	24
Skopje	−0.360	0.094	0.128	0.256	−0.656	80	52	58	76	81
Sofia	−0.201	−0.150	0.062	0.361	−0.329	71	74	65	66	69
Stockholm	0.058	0.022	0.407	0.500	0.313	56	57	13	38	11
Strasbourg	0.322	0.366	0.235	0.424	0.238	14	16	41	55	18
Tallinn	0.284	0.234	0.484	0.666	−0.281	21	37	4	11	64
Tirana	0.003	0.192	−0.036	0.486	−0.536	63	46	69	40	79
Turin	−0.343	−0.251	−0.315	0.258	−0.130	79	77	77	75	53
Tyneside conurbation	0.260	0.406	0.262	0.443	0.219	26	12	35	50	20
Valletta	0.223	0.400	0.418	0.581	0.303	30	14	10	23	12
Verona	−0.119	−0.117	0.174	0.420	−0.315	68	71	47	56	67
Vilnius	0.059	0.219	0.111	0.428	−0.065	55	41	60	53	48
Warszawa	0.145	−0.031	0.085	0.589	0.032	41	67	63	21	42
Wien	0.445	0.358	0.441	0.700	0.389	5	17	8	6	7
Zagreb	−0.341	−0.261	−0.211	0.336	−0.661	78	79	76	69	83
Zurich	0.610	0.517	0.583	0.718	0.501	1	7	1	5	3

**Table A7:** Values of IFTOPSIS coefficients for cities.

City	IFT__me2_	IFT__me3_	IFT__mh2_	IFT__mh3_	IFT__ae2_	IFT__ae3_	IFT__ah2_	IFT__ah3_
Aalborg	0.835	0.824	0.873	0.850	0.737	0.777	0.751	0.797
Amsterdam	0.638	0.628	0.658	0.643	0.623	0.688	0.632	0.711
Ankara	0.621	0.616	0.627	0.603	0.612	0.700	0.615	0.731
Antalya	0.747	0.734	0.784	0.743	0.693	0.755	0.702	0.786
Antwerpen	0.669	0.656	0.680	0.654	0.638	0.699	0.644	0.719
Athina	0.240	0.245	0.218	0.229	0.396	0.550	0.388	0.566
Barcelona	0.493	0.490	0.495	0.480	0.540	0.651	0.542	0.684
Belfast	0.654	0.647	0.666	0.652	0.629	0.691	0.636	0.709
Beograd	0.247	0.249	0.230	0.235	0.405	0.553	0.395	0.570
Berlin	0.435	0.437	0.415	0.430	0.498	0.594	0.498	0.612
Białystok	0.725	0.704	0.749	0.705	0.674	0.732	0.683	0.758
Bologna	0.556	0.552	0.562	0.538	0.575	0.671	0.579	0.701
Bordeaux	0.672	0.657	0.677	0.649	0.638	0.707	0.643	0.729
Braga	0.535	0.530	0.550	0.532	0.567	0.660	0.572	0.689
Bratislava	0.543	0.539	0.549	0.550	0.566	0.647	0.572	0.671
Bruxelles	0.718	0.702	0.741	0.701	0.673	0.737	0.678	0.764
Bucharest	0.444	0.443	0.432	0.432	0.506	0.610	0.507	0.634
Budapest	0.569	0.560	0.585	0.579	0.585	0.652	0.592	0.671
Burgas	0.520	0.516	0.544	0.524	0.563	0.657	0.569	0.690
Cardiff	0.779	0.761	0.790	0.761	0.697	0.742	0.705	0.758
Cluj-Napoca	0.579	0.571	0.615	0.594	0.594	0.662	0.608	0.691
Diyarbakir	0.457	0.456	0.442	0.437	0.512	0.634	0.512	0.663
Dortmund	0.483	0.478	0.480	0.471	0.532	0.632	0.533	0.655
Dublin	0.738	0.730	0.758	0.739	0.679	0.733	0.688	0.753
Essen	0.575	0.561	0.577	0.561	0.581	0.651	0.587	0.673
Gdańsk	0.688	0.671	0.715	0.679	0.654	0.718	0.664	0.745
Genève	0.699	0.690	0.739	0.719	0.665	0.722	0.677	0.747
Glasgow	0.625	0.620	0.628	0.620	0.611	0.680	0.616	0.697
Graz	0.807	0.790	0.831	0.790	0.720	0.771	0.728	0.795
Groningen	0.752	0.726	0.796	0.750	0.692	0.729	0.709	0.750
Hamburg	0.647	0.629	0.657	0.632	0.624	0.687	0.632	0.709
Helsinki	0.597	0.599	0.598	0.606	0.593	0.671	0.599	0.696
Irakleio	0.288	0.293	0.284	0.296	0.433	0.574	0.425	0.598
Istanbul	0.518	0.516	0.528	0.512	0.557	0.658	0.560	0.688
København	0.803	0.795	0.837	0.819	0.718	0.765	0.731	0.788
Košice	0.596	0.586	0.625	0.603	0.604	0.676	0.614	0.704
Kraków	0.606	0.593	0.620	0.600	0.603	0.675	0.611	0.700
Lefkosia	0.581	0.578	0.621	0.611	0.600	0.686	0.611	0.725
Leipzig	0.668	0.638	0.687	0.656	0.638	0.686	0.648	0.705
Liège	0.671	0.655	0.691	0.662	0.643	0.702	0.650	0.724
Lille	0.611	0.597	0.613	0.589	0.603	0.679	0.607	0.701
Lisboa	0.375	0.374	0.382	0.383	0.481	0.600	0.479	0.624
Ljubljana	0.592	0.589	0.624	0.613	0.602	0.673	0.613	0.700
London	0.675	0.667	0.688	0.675	0.641	0.705	0.649	0.726
Luxembourg	0.827	0.817	0.847	0.816	0.731	0.785	0.737	0.811
Madrid	0.559	0.553	0.557	0.538	0.574	0.665	0.576	0.689
Málaga	0.579	0.576	0.582	0.575	0.587	0.669	0.590	0.690
Malmö	0.676	0.674	0.687	0.684	0.639	0.703	0.648	0.725
Manchester	0.757	0.756	0.774	0.771	0.688	0.744	0.696	0.765
Marseille	0.458	0.454	0.446	0.446	0.514	0.618	0.515	0.639
Miskolc	0.613	0.601	0.645	0.622	0.612	0.664	0.625	0.685
Munich	0.676	0.657	0.691	0.673	0.641	0.697	0.650	0.717
Naples	0.175	0.176	0.157	0.157	0.362	0.537	0.355	0.549
Oslo	0.527	0.530	0.521	0.524	0.551	0.624	0.556	0.640
Ostrava	0.562	0.559	0.594	0.588	0.585	0.662	0.597	0.692
Oulu	0.636	0.635	0.631	0.626	0.614	0.693	0.617	0.716
Oviedo	0.458	0.456	0.450	0.444	0.517	0.628	0.517	0.651
Palermo	0.071	0.079	0.038	0.050	0.305	0.509	0.289	0.513
Paris	0.608	0.597	0.612	0.585	0.603	0.685	0.606	0.710
Piatra Neamt	0.556	0.549	0.570	0.550	0.575	0.649	0.583	0.675
Podgorica	0.442	0.440	0.439	0.432	0.510	0.618	0.511	0.646
Praha	0.548	0.544	0.570	0.565	0.573	0.641	0.583	0.664
Rennes	0.757	0.737	0.768	0.725	0.688	0.748	0.693	0.772
Reykjavík	0.388	0.398	0.390	0.414	0.485	0.597	0.483	0.618
Riga	0.334	0.338	0.314	0.332	0.447	0.577	0.441	0.598
Rome	0.058	0.070	0.045	0.062	0.311	0.511	0.293	0.515
Rostock	0.719	0.696	0.739	0.707	0.667	0.716	0.677	0.736
Rotterdam	0.666	0.644	0.683	0.658	0.638	0.697	0.646	0.719
Skopje	0.341	0.340	0.322	0.317	0.452	0.585	0.446	0.612
Sofia	0.378	0.385	0.373	0.390	0.476	0.583	0.474	0.600
Stockholm	0.645	0.636	0.654	0.636	0.621	0.678	0.630	0.695
Strasbourg	0.700	0.683	0.706	0.672	0.655	0.720	0.659	0.742
Tallinn	0.624	0.620	0.670	0.652	0.622	0.679	0.639	0.705
Tirana	0.432	0.432	0.439	0.435	0.510	0.628	0.511	0.664
Turin	0.294	0.296	0.279	0.280	0.426	0.570	0.422	0.588
Tyneside conurbation	0.699	0.685	0.707	0.684	0.653	0.706	0.659	0.721
Valletta	0.742	0.711	0.767	0.728	0.682	0.720	0.693	0.736
Verona	0.426	0.425	0.427	0.422	0.504	0.618	0.504	0.645
Vilnius	0.551	0.549	0.555	0.557	0.572	0.654	0.575	0.673
Warszawa	0.556	0.546	0.568	0.547	0.576	0.660	0.582	0.687
Wien	0.829	0.816	0.841	0.809	0.727	0.777	0.733	0.798
Zagreb	0.234	0.246	0.214	0.230	0.399	0.543	0.386	0.557
Zurich	0.920	0.895	0.948	0.911	0.785	0.814	0.793	0.827

**Table A8:** Rank of IFTOPSIS coefficients for cities.

City	IFT__me2_	IFT__me3_	IFT__mh2_	IFT__mh3_	IFT__ae2_	IFT__ae3_	IFT__ah2_	IFT__ah3_
Aalborg	2	2	2	2	2	4	2	4
Amsterdam	32	33	31	31	31	31	31	31
Ankara	36	36	37	41	35	25	37	20
Antalya	11	10	9	10	8	7	9	7
Antwerpen	26	25	27	27	28	26	27	27
Athina	79	80	79	80	80	79	79	79
Barcelona	61	61	61	61	61	56	61	54
Belfast	29	27	30	28	29	30	30	33
Beograd	78	78	78	78	78	78	78	78
Berlin	68	68	70	69	70	72	70	72
Białystok	14	14	14	16	14	13	14	12
Bologna	52	51	53	55	51	44	53	38
Bordeaux	24	23	28	30	26	20	28	21
Braga	57	57	56	57	56	50	56	49
Bratislava	56	56	57	52	57	59	57	59
Bruxelles	16	15	15	17	15	11	15	10
Bucharest	66	66	68	68	68	69	68	69
Budapest	48	48	47	47	48	55	47	58
Burgas	59	59	58	59	58	53	58	47
Cardiff	7	7	8	8	7	10	8	11
Cluj-Napoca	46	46	42	43	44	49	42	46
Diyarbakir	65	64	65	65	65	61	65	62
Dortmund	62	62	62	62	62	62	62	63
Dublin	13	11	13	11	13	12	13	13
Essen	47	47	49	50	49	57	49	57
Gdańsk	20	21	18	20	19	18	18	16
Genève	19	17	17	14	17	15	17	15
Glasgow	34	35	36	36	37	36	36	42
Graz	5	6	6	6	5	5	6	5
Groningen	10	12	7	9	9	14	7	14
Hamburg	30	32	32	33	30	32	32	34
Helsinki	41	38	45	39	45	43	45	43
Irakleio	77	77	76	76	76	76	76	76
Istanbul	60	60	59	60	59	52	59	51
København	6	5	5	3	6	6	5	6
Košice	42	43	38	40	38	40	38	37
Kraków	40	41	41	42	40	41	41	40
Lefkosia	44	44	40	38	43	33	40	24
Leipzig	27	29	25	26	25	34	25	35
Liège	25	26	22	24	21	24	22	25
Lille	38	40	43	44	39	38	43	39
Lisboa	73	73	72	73	72	70	72	70
Ljubljana	43	42	39	37	42	42	39	41
London	23	22	23	21	22	22	23	22
Luxembourg	4	3	3	4	3	2	3	2
Madrid	50	50	54	56	53	46	54	50
Málaga	45	45	48	48	46	45	48	48
Malmö	21	20	24	19	24	23	24	23
Manchester	8	8	10	7	11	9	10	9
Marseille	64	65	64	63	64	67	64	68
Miskolc	37	37	34	35	36	47	34	53
Munich	22	24	21	22	23	27	21	29
Naples	81	81	81	81	81	81	81	81
Oslo	58	58	60	58	60	65	60	67
Ostrava	49	49	46	45	47	48	46	45
Oulu	33	31	35	34	34	29	35	30
Oviedo	63	63	63	64	63	63	63	64
Palermo	82	82	83	83	83	83	83	83
Paris	39	39	44	46	41	35	44	32
Piatra Neamt	53	53	50	53	52	58	50	55
Podgorica	67	67	67	67	67	68	67	65
Praha	55	55	51	49	54	60	51	61
Rennes	9	9	11	13	10	8	11	8
Reykjavík	71	71	71	71	71	71	71	71
Riga	75	75	75	74	75	75	75	75
Rome	83	83	82	82	82	82	82	82
Rostock	15	16	16	15	16	19	16	19
Rotterdam	28	28	26	25	27	28	26	28
Skopje	74	74	74	75	74	73	74	73
Sofia	72	72	73	72	73	74	73	74
Stockholm	31	30	33	32	33	39	33	44
Strasbourg	17	19	20	23	18	17	20	17
Tallinn	35	34	29	29	32	37	29	36
Tirana	69	69	66	66	66	64	66	60
Turin	76	76	77	77	77	77	77	77
Tyneside conurbation	18	18	19	18	20	21	19	26
Valletta	12	13	12	12	12	16	12	18
Verona	70	70	69	70	69	66	69	66
Vilnius	54	52	55	51	55	54	55	56
Warszawa	51	54	52	54	50	51	52	52
Wien	3	4	4	5	4	3	4	3
Zagreb	80	79	80	79	79	80	80	80
Zurich	1	1	1	1	1	1	1	1

## References

[1] Greco S., Ehrgott M., Figueira J.R. (2016). Multiple Criteria Decision Analysis: State of the Art Surveys.

[2] Lu K., Liao H., Zavadskas E.K. (2021). An Overview of Fuzzy Techniques in Supply Chain Management: Bibliometrics, Methodologies, Applications and Future Directions. Technol. Econ. Dev. Econ..

[3] Mardani A., Jusoh A., Zavadskas E.K. (2015). Fuzzy Multiple Criteria Decision-Making Techniques and Applications–Two Decades Review from 1994 to 2014. Expert Syst. Appl..

[4] Greco S., Ishizaka A., Tasiou M., Torrisi G. (2019). On the Methodological Framework of Composite Indices: A Review of the Issues of Weighting, Aggregation, and Robustness. Soc. Indic. Res..

[5] Munda G., Nardo M. (2005). Constructing Consistent Composite Indicators: The Issue of Weights.

[6] Saisana M., Tarantola S. (2002). State-of-the-Art Report on Current Methodologies and Practices for Composite Indicator Development.

[7] Hellwig Z. (1968). Zastosowanie Metody Taksonomicznej Do Typologicznego Podziału Krajów Ze Względu Na Poziom Ich Rozwoju Oraz Zasoby i Strukturę Wykwalifikowanych Kadr. Przegląd Stat..

[8] Hellwig Z., Gostkowski Z. (1972). Procedure of Evaluating High-Level Manpower Data and Typology of Countries by Means of the Taxonomic Method. Towards a System of Human Resources Indicators for Less Developed Countries.

[9] Hellwig Z., Gostkowski Z. (1968). On the Optimal Choice of Predictors. Study VI. Toward a System of Quantitative Indicators of Components of Human Resources Development.

[10] Baster N. (1972). Measuring Development: The Role and Adequacy of Development Indicators.

[11] Di Domizio M. (2008). The Competitive Balance in the Italian Football League: A Taxonomic Approach.

[12] Pawlas I. (2016). Economic Picture of the Enlarged European Union in the Light of Taxonomic Research. Proc. MAC-EMM.

[13] Reiff M., Surmanová K., Balcerzak A.P., Pietrzak M.B. (2016). Multiple Criteria Analysis of European Union Agriculture. J. Int. Stud..

[14] Roszkowska E., Filipowicz-Chomko M. (2020). Measuring Sustainable Development Using an Extended Hellwig Method: A Case Study of Education. Soc. Indic. Res..

[15] Jefmański B., Dudek A., Appenzeller D. (2016). Syntetyczna Miara Rozwoju Hellwiga dla trójkątnych liczb rozmytych [Hellwig’s Measure of Development for Triangular Fuzzy Numbers]. Matematyka i Informatyka na Usługach Ekonomii. Wybrane Problemy Modelowania i Prognozowania Zjawisk Gospodarczych.

[16] Łuczak A., Wysocki F. (2007). Rozmyta Wielokryterialna Metoda Hellwiga Porządkowania Liniowego Obiektów. Pr. Nauk. Akad. Ekon. Wrocławiu Taksonomia.

[17] Wysocki F. (2010). Metody Taksonomiczne w Rozpoznawaniu Typów Ekonomicznych Rolnictwa i Obszarów Wiejskich [Taxonomic Methods in Recognizing Economic Types of Agriculture and Rural Areas].

[18] Jefmański B., Jajuga K., Batóg J., Walesiak M. (2020). Intuitionistic Fuzzy Synthetic Measure for Ordinal Data. Classification and Data Analysis, Proceedings of the Conference of the Section on Classification and Data Analysis of the Polish Statistical Association, Szczecin, Poland, 18–20 September 2019.

[19] Roszkowska E., Kusterka-Jefmańska M., Jefmański B. (2021). Intuitionistic Fuzzy TOPSIS as a Method for Assessing Socioeconomic Phenomena on the Basis of Survey Data. Entropy.

[20] Roszkowska E. (2021). The Intuitionistic Fuzzy Framework for Evaluation and Rank Ordering the Negotiation Offers. Intelligent and Fuzzy Techniques for Emerging Conditions and Digital Transformation, Proceedings of the International Conference on Intelligent and Fuzzy Systems, Istanbul, Turkey, 24–26 August 2021.

[21] Roszkowska E., Jefmański B. (2021). Interval-Valued Intuitionistic Fuzzy Synthetic Measure (I-VIFSM) Based on Hellwig’s Approach in the Analysis of Survey Data. Mathematics.

[22] Lindén D., Cinelli M., Spada M., Becker W., Gasser P., Burgherr P. (2021). A Framework Based on Statistical Analysis and Stakeholders’ Preferences to Inform Weighting in Composite Indicators. Environ. Model. Softw..

[23] Atanassov K.T. (1986). Intuitionistic Fuzzy Sets. Fuzzy Sets Syst..

[24] Zadeh L.A. (1965). Information and Control. Fuzzy Sets.

[25] Atanassov K.T. (1999). Intuitionistic Fuzzy Sets. Theory and Applications.

[26] Shen F., Ma X., Li Z., Xu Z., Cai D. (2018). An Extended Intuitionistic Fuzzy TOPSIS Method Based on a New Distance Measure with an Application to Credit Risk Evaluation. Inf. Sci..

[27] Xu Z. (2007). Intuitionistic Fuzzy Aggregation Operators. IEEE Trans. Fuzzy Syst..

[28] Szmidt E. (2014). Distances and Similarities in Intuitionistic Fuzzy Sets.

[29] Chen S.-M., Tan J.-M. (1994). Handling Multicriteria Fuzzy Decision-Making Problems Based on Vague Set Theory. Fuzzy Sets Syst..

[30] Hong D.H., Choi C.-H. (2000). Multicriteria Fuzzy Decision-Making Problems Based on Vague Set Theory. Fuzzy Sets Syst..

[31] Xu Z., Yager R.R. (2006). Some Geometric Aggregation Operators Based on Intuitionistic Fuzzy Sets. Int. J. Gen. Syst..

[32] Maggino F., Ruviglioni E. Obtaining Weights: From Objective to Subjective Approaches in View of More Participative Methods in the Construction of Composite Indicators. Proceedings of the NTTS: New Techniques and Technologies for Statistics.

[33] (2020). Report on the Quality of Life in European Cities.

[34] Hwang C.-L., Yoon K. (1981). Methods for Multiple Attribute Decision Making.

[35] Behzadian M., Khanmohammadi Otaghsara S., Yazdani M., Ignatius J. (2012). A State-of the-Art Survey of TOPSIS Applications. Expert Syst. Appl..

[36] Palczewski K., Sałabun W. (2019). The Fuzzy TOPSIS Applications in the Last Decade. Procedia Comput. Sci..

